# Mood, Activity Participation, and Leisure Engagement Satisfaction (MAPLES): results from a randomised controlled pilot feasibility trial for low mood in acquired brain injury

**DOI:** 10.1186/s12916-023-03128-7

**Published:** 2023-11-16

**Authors:** Andrea Kusec, Fionnuala C. Murphy, Polly V. Peers, Ron Bennett, Estela Carmona, Aleksandra Korbacz, Cara Lawrence, Emma Cameron, Andrew Bateman, Peter Watson, Judith Allanson, Pieter duToit, Tom Manly

**Affiliations:** 1 MRC Cognition and Brain Sciences Unit, University of Cambridge, Cambridge, UK; 2 Nuffield Department of Clinical Neurosciences, University of Oxford, Oxford, UK; 3 Patient and Public Involvement Representative, University of Cambridge, Cambridge, UK; 4 School of Allied Health, Anglia Ruskin University, Cambridge, UK; 5 University College London Hospitals NHS Foundation Trust, London, UK; 6 School of Health and Social Care, University of Essex, Colchester, UK; 7 Cambridge University Hospitals NHS Foundation Trust, Cambridge, UK; 8 The Disabilities Trust, Fen House, Ely, UK

**Keywords:** Acquired brain injury, Traumatic brain injury, Depression, Executive function, Neuropsychological rehabilitation, Randomised trial, Feasibility trial

## Abstract

**Background:**

Acquired brain injury (ABI) is linked to increased depression risk. Existing therapies for depression in ABI (e.g., cognitive behavioural therapy) have mixed efficacy. Behavioural activation (BA), an intervention that encourages engaging in positively reinforcing activities, shows promise. The primary aims were to assess feasibility, acceptability, and potential efficacy of two 8-week BA groups.

**Methods:**

Adults (≥ 18 years) recruited from local ABI services, charities, and self-referral via social media were randomised to condition. The Activity Planning group (AP; “traditional” BA) trained participants to plan reinforcing activities over 8 weeks. The Activity Engagement group (AE; “experiential” BA) encouraged engagement in positive activities within session only. Both BA groups were compared to an 8-week Waitlist group (WL). The primary outcomes, feasibility and acceptability, were assessed via recruitment, retention, attendance, and qualitative feedback on groups. The secondary outcome, potential efficacy, was assessed via blinded assessments of self-reported activity levels, depression, and anxiety (at pre- and post-intervention and 1 month follow-up) and were compared across trial arms. Data were collected in-person and remotely due to COVID-19.

**Results:**

*N* = 60 participants were randomised to AP (randomised *n* = 22; total *n* = 29), AE (randomised *n* = 22; total *n* = 28), or re-randomised following WL (total *n* = 16). Whether in-person or remote, AP and AE were rated as similarly enjoyable and helpful. In exploring efficacy, 58.33% of AP members had clinically meaningful activity level improvements, relative to 50% AE and 38.5% WL. Both AP and AE groups had depression reductions relative to WL, but only AP participants demonstrated anxiety reductions relative to AE and WL. AP participants noted benefits of learning strategies to increase activities and learning from other group members. AE participants valued social discussion and choice in selecting in-session activities.

**Conclusions:**

Both in-person and remote group BA were feasible and acceptable in ABI. Though both traditional and experiential BA may be effective, these may have different mechanisms.

**Trial registration:**

Clinicaltrials.gov, NCT03874650. Protocol version 2.3, May 26 2020.

**Supplementary Information:**

The online version contains supplementary material available at 10.1186/s12916-023-03128-7.

## Background

An acquired brain injury (ABI) can result from a blow to the head (traumatic brain injury; TBI), a blockage to the brain’s blood supply (stroke, aneurysm), lack of oxygen to the brain (anoxia, hypoxia), viral infections (encephalitis), or damage from a resected brain tumour. This definition excludes brain abnormalities arising from a congenital disorder, developmental disability, or progressive neurological conditions [1].

ABI markedly increases risk of a mood disorder that can begin weeks, months, or even years post-injury [2–4]. Whilst cognitive behavioural therapy (CBT) is often considered the intervention of choice for depression in the general population, CBT studies in ABI tend to have more mixed outcomes and lower effect sizes [5, 6]. This could be due to the cognitive demands of CBT on capacities such as meta-awareness, memory, and mental flexibility that may be compromised by ABI which may not be accommodated for in CBT delivered within general psychological services [5, 7].

A promising alternative intervention is behavioural activation (BA). BA theory is based on the premise that people with low mood tend to engage in fewer positively reinforcing activities (e.g., because of low motivation, inability to anticipate positive outcomes, or anxious avoidance). Although this may feel protective of mood, the counter-productive consequence of this can reduce positive reinforcement, and in turn further reduce mood, establishing a negative cycle of worsening depression [8]. The relatively simple idea of BA is to intervene at the behavioural level by encouraging individuals to plan and engage in positively reinforcing activities, rather than trying to directly address mood [9]. BA has been shown to be as effective as CBT and anti-depressant medication in reducing depression in the non-ABI population, showing strong effects both individually (*d* = 0.78) and in group settings (*d* = 0.74) [10–12]. BA is a particularly promising approach in ABI given its conceptual simplicity and its lowered demands on cognitive abilities (e.g., poor memory and planning difficulties). In line with this, systematic reviews have reported preliminary support for one-to-one BA therapy in stroke [13, 14] and in progressive neurological populations (*d* = 0.24 to 1.70) [15].

The current study builds on the promising BA research in neurological populations by being the first to evaluate *group* BA interventions in the wider ABI population (i.e., including—but not limited to—those with stroke). If shown to be effective, group approaches have clear economic advantages [16–18] and, indeed, in some cases can show enhanced intervention efficacy, e.g., due to the added benefits of peer support [19, 20].

Although *relatively* less cognitively demanding compared to CBT, BA nevertheless has significant cognitive demands. If an individual has difficulties planning activities, forgets intentions and/or struggles to recall the positive aspects of activities, BA’s efficacy could be undermined. Accordingly, in developing BA for ABI, we incorporated components of executive function interventions in ABI that seek to help people better manage attention, planning, and intention execution [21–24]. Components included breaking complex goals down into smaller steps (“task splitting”), identifying and mitigating causes of distraction, and preventing automatic behaviours interfering with activity completion. Though adaptations to BA are often used clinically (e.g., external memory aids, involvement of family members), only one study to date has investigated augmented BA with intention implementation support and in TBI only [25].

Despite the inclusion of goal management techniques, it is still possible that there are individuals with greater cognitive problems from ABI that struggle to remember and implement intentions in everyday life, potentially undermining BA efficacy. For example, in “traditional” BA sessions, therapists work with clients to identify potentially positive activities that the client can independently complete between sessions. The outcomes and their effect on mood are then discussed at subsequent sessions, hopefully reinforcing the positive activity engagement—mood link. The rationale is that experience completing positively reinforcing activities will produce better maintained and generalised gains after therapy ends, relative to BA psychoeducation alone. If an individual struggles with these processes even with the incorporation of cognitive strategies to support activity planning, facilitating potentially reinforcing activities *within* group therapy sessions could be a more effective BA approach (i.e., removing the need for planning, engaging in, and reporting back on activities). Positive experiences in such a group might implicitly challenge underlying counter-productive beliefs and increase seeking of positive activities in everyday life.

While activity-based peer support groups are already offered by, e.g., ABI charities, they have rarely, if ever, been formally compared to another or to no intervention [19]. Peer support groups are recommended within the UK [19] and are viewed as beneficial by ABI survivors [25]. In within-group research designs, peer support groups have been shown to improve well-being and quality of life in ABI [26, 27] and have extended benefits in gaining understanding from others and increasing hopefulness and injury acceptance [28, 29]. It is likely that such groups are in themselves a source of positive reinforcement and mood improvement, in line with the central tenet of BA theory that reductions in depression symptoms are due to increases in positive activity engagement.

Relative to “traditional” BA groups, activity-based peer support groups may be viewed as more acceptable by ABI survivors, given their greater focus on socialisation. However, only “traditional” BA incorporates formal psychoeducation on the importance of the activity–mood relationship and a range of self-management techniques to increase independence in daily activities. The increased focus on developing independence in scheduling and maintaining positive activities may encourage greater long-term mood improvements relative to peer support groups where positive reinforcement is predominantly within session.

Taken together, here we compared a “Traditional” BA group (Activity Planning group, “AP”) to an “Experiential” BA group (Activity Engagement group, “AE”) in which participants engaged in socialisation, quizzes, and crafts and in which a positive social atmosphere was encouraged.

### Primary objectives

The primary objective of the Mood, Activity Participation, and Leisure Engagement Satisfaction (MAPLES) trial was to determine the feasibility and acceptability of two activity-based group interventions in adults with ABI with low mood and/or activity levels, as per our trial protocol [30]. Feasibility and acceptability were assessed via participant retention from baseline to 1-month post-intervention, acceptability of group sessions and assessments, and qualitative interview feedback. Although originally planned to occur completely in-person, the COVID-19 pandemic and resulting social distancing regulations meant that the study pivoted to online delivery in March 2020. This offered the opportunity to compare the two forms of delivery. Therefore, we report combined results and, where appropriate, in-person and online results separately.

### Secondary objectives

The secondary objective was to explore whether *either* Traditional (AP) or Experiential (AE) Behavioural Activation leads to changes in activity levels and related outcomes, including depression, anxiety, post-traumatic stress, motivation, participation, and sense of control. To control for repeated exposure to assessments, and to ensure all participants were able to receive a potentially helpful intervention, AP and AE participants were compared to a waitlist control (WL) condition in ABI as per Kahan et al. [31].

## Methods

### Trial design

MAPLES is a 1:1:1 parallel arm randomised controlled trial with nested qualitative research. A CONSORT checklist is in Additional File 1*.*

### Research ethics approval and consent

The UK National Health Service Health Research Authority (REC reference: 18/EE/0305) provided ethical approval. The study was registered at clinicaltrials.gov on March 12, 2019 (NCT03874650). All participants provided informed consent prior to participation. Approval to conduct groups online was obtained June 5, 2020.

### Study setting

Participants were recruited from two NHS sites in Cambridgeshire, UK. In-person sessions were held in NHS sites or at the MRC Cognition and Brain Sciences Unit. Following the onset of COVID-19, non-COVID research was halted across all NHS Trusts. Recruitment expanded to ABI research panels, ABI charities, and self-referrals (ABIs verified by research team via medical record access) via social media. These sessions were delivered online using videoconferencing.

### Eligibility criteria

Inclusion criteria:

1. Diagnosis of an ABI^1^
2.  ≥ 18 years old
3. Ability to speak and comprehend English
4. Minimum 3 months post-ABI
5. Low mood/reduced activity level, identified by either:
  - Scoring 7 or above on the Hospital Anxiety and Depression Scale-Depression subscale (HADS-D [32].)
  - Clinician report (i.e., through clinician’s own administration of the HADS-D within the past 3 months or clinical interview indicating a client has low mood/could benefit emotionally from increased activity level).

Exclusion criteria:

1. Incapable of attending to and/or understanding the intervention materials
2. Diagnosis of dementia or other neurodegenerative disorder
3. Unstable psychotropic medication (i.e., started/changed medications in past 6 weeks)
4. Active suicidality (i.e., attempted suicide in past 3 months, currently self-harming, and/or had suicidal intentions for near future)

Individuals with mild traumatic brain injury were not excluded from accessing the trial provided a confirmation of diagnosis from medical records. In the protocol [30], we additionally excluded participants who were currently undergoing or due to undergo a psychological intervention during the trial. This criterion was removed in agreement with the Steering Committee as, in practice, excluding potentially eligible participants who met with a therapist infrequently (e.g., once every 3 months) was a recruitment barrier. Differences in proportions of those receiving any type of therapeutic input across study arms was assessed.

### Interventions

Both groups met for approximately 1.5 h once weekly over 8 weeks. Group sizes ranged from 3 to 6 individuals. WL participants received no intervention for an 8-week period between first and second assessments. The interventions are described briefly below. Detailed descriptions are in the study protocol [30].

### Facilitator training

Both groups were facilitated by AK1. AK1 received intervention training (approximately 6 h) and regular supervisions from senior research team members, which included a registered clinical psychologist (TM).

### Activity Planning (AP) group

The AP group was designed based on typical BA interventions (see Kanter et al. [33]) and interviews with ABI participants and carers [34]. AP sessions centred on increasing engagement in meaningful and positive activities. Participants were instructed to “task split” planned activities into steps and monitor their mood and activities to help identify connections between the two [8, 35]. Participants identified counter-productive avoidance patterns, including distraction and goal neglect (not completing a stated intention [36]), and practiced strategies to overcome these [21, 37]. Session content is described in brief in Table 1.

**Table 1:** MAPLES BA groups session overview. The Activity Planning group followed a structured, linear progression of BA with between-session activities, while the Activity Engagement group followed a non-linear structure where participants were free to use session time to engage in a variety of activities or socialise with group members

	**Activity Planning Group**	**Activity Engagement Group**
Session 1	Participants are provided with psychoeducation of BA, the mood-activity relationship, and executive functioning difficulties after ABI. Participants are introduced to mood monitoring and monitoring lapses in attention	Participants are provided with overview of group was to use session time to take part in enjoyable or meaningful activities. Participants take part in icebreakers and were encouraged to provide suggestions for activities to complete
Session 2	Participants discuss monitoring homework and identify activities that align with personal goals and values. Participants schedule their first activity using task splitting	Participants were re-offered opportunity to suggest session activities. Participants complete in-session activity and social engagement is encouraged
Session 3	Participants review planned activity homework and discuss impact on mood (henceforth “review homework”). Participants are provided with psychoeducation on how activities become habitual and identify personal triggers that interfere with activity completion. Participants schedule another activity using task splitting	Participants were re-offered opportunity to suggest session activities. Participants complete in-session activity and social engagement is encouraged
Session 4	Participants review homework, and Week 2 values and goals. Participants discuss whether activities currently in their routine negatively contribute to mood and whether these can be altered. Participants schedule another activity using task splitting	Participants were re-offered opportunity to suggest session activities. Participants complete in-session activity and social engagement is encouraged
Session 5	Participants review homework. Participants are provided with psychoeducation on identifying avoidance patterns to activities and how to prevent task distraction. Participants schedule another activity using task splitting	Participants were re-offered opportunity to suggest session activities. Participants complete in-session activity and social engagement is encouraged
Session 6	Participants review homework. Participants are provided with psychoeducation on benefits of continuously increasing activity levels and warning signs of fatigue when increasing activities. Participants schedule another activity using task splitting and raise activity level further (e.g., in duration and/or frequency)	Participants were re-offered opportunity to suggest session activities. Participants complete in-session activity and social engagement is encouraged
Session 7	Participants review homework. Participants are provided with psychoeducation on benefits of social relationships to positive mood. Participants identify barriers to planning social activities and role play initiating social activities. Participants schedule another activity using task splitting, with encouragement to plan a social activity	Participants were re-offered opportunity to suggest session activities. Participants complete in-session activity and social engagement is encouraged
Session 8	Participants review homework. Participants review content from past 7 sessions and outline “take home” messages. Participants identify personal triggers to reducing activity levels and create strategies to overcome triggers. Participants schedule another activity using task splitting, with encouragement to continue engaging in activities beyond group end	Participants were re-offered opportunity to suggest session activities. Participants complete in-session activity and social engagement is encouraged

### Activity Engagement (AE) group

AE participants received no specific encouragement to increase activity engagement between sessions or to overcome barriers to activity participation in everyday life. Rather, they were told that they would complete potentially rewarding activities during sessions. At the start of the 8 weeks, participants were told that one approach to increasing activities was to “learn by doing” and as part of the group they will take part in potentially reinforcing/enjoyable activities. Participants were offered suggestions, such as card games and “pub quizzes,” and were encouraged to suggest activities (including repeating an activity from a previous session). Session content was based on typical ABI charity activities. Over the course of the 8 sessions, participants were encouraged to jointly discuss which activities they would like to complete.

### Waitlist (WL) control group

One-third of participants were first assigned to WL. At the end of the 8-week waiting period, participants completed a second baseline assessment and were immediately re-randomised into either AP or AE. To produce unbiased estimates, the WL design followed the recommendations of Kahan et al. [31], where re-randomisations were (1) conducted only after the WL period was completed and (2) conducted independently from the initial randomisation sequence. Further, potential effects of either BA group were assumed to have comparable effects across randomisations [31].

### Intervention fidelity

Group sessions were audio recorded to assess intervention fidelity. Two research assistants not conducting the intervention retrospectively listened to 25% (42 h) of sessions. AP group fidelity was assessed in terms of whether intervention components were delivered. Given the nature of the AE group, general principles (e.g., not discussing planning activities outside of sessions) rather than specific content were used to evaluate fidelity (see Additional File 2: Document S1 for Fidelity checklists). Research assistants followed the below steps [38]:

1. Listen to audiorecording and assess fidelity together;
2. Listen to audiorecording together and assess fidelity separately;
3. Listen to audiorecording separately and assess fidelity separately.

Following steps 2 and 3, raters compared their scores and resolved discrepancies, if any. Sufficient agreement was considered reached once raters had less than 10% discrepant items, after which ratings were completed independently. All rating checklist items were summed and given percent scores per checklist, per group. Percent scores were then averaged for a total estimate.

### Primary objective

Feasibility and acceptability were determined based on targets set out in the accompanying protocol paper [30]:

### Quantitative targets

1. Minimum of 9, maximum 18 participants recruited (i.e., minimum 3 people in all groups in parallel) per cohort of trial arms
2. Attrition < 20% across the three trial arms
3. Average attendance of at least 5/8 sessions within AP and AE groups

### Mixed-methods targets

4) Sufficiently positive ratings of groups in the post-study questionnaire
5) Minimal barriers to attendance and engagement in groups reported relative to benefits discussed in the qualitative data.

### Secondary objective—primary outcome measure

Because the key aim of BA is to increase engagement in activities, the *Behavioural Activation for Depression Scale* (BADS [39, 40]) was selected as the primary efficacy outcome measure. The BADS is a 25-item measure of activation and avoidance behaviours underlying depression. Higher scores indicate greater activity engagement (“behavioural activation”).

### Participant timeline

Participants were recruited for 18 months and enrolled in 7 successive cohorts or “waves”.

### Sample size

The primary objective was to determine feasibility and acceptability. Approximately 20 participants per arm (total 60) was considered sufficient to balance between determining feasibility and exploring treatment effects [41].

### Recruitment

Participants recruited through any source were first given invitation letters, providing a brief study summary. Interested individuals were screened to determine eligibility. If eligible, an appointment was made to take informed consent and conduct the baseline assessment.

### Allocation—sequence generation

Pre-determined block randomisation with blocks of varying length was generated by the trial statistician and was unknown to the rest of the team. Participants first assigned to WL were re-randomised using pre-determined block randomisation.

### Allocation concealment mechanism, blinding, and implementation

The statistician placed each condition allocation into separate sealed opaque envelopes. Envelopes were taken in sequence to baseline assessments and opened by the first author immediately *after* the assessment was complete; hence, these were conducted blind to condition. This was repeated for re-randomising WL participants. To mitigate potential delays between the baseline assessment and interventions beginning and maximise attendance, participants were informed of their allocation immediately, and potentially available dates and times (e.g., preference for Thursdays) collected.

Quantitative assessments at Times 2 and 3 were conducted by condition-blinded research assistants. It was not possible for researchers conducting qualitative interviews (that discussed specific group experiences) to be blind to condition.

### Data collection methods

Depending on COVID-19 restrictions, participants gave informed consent in-person or via videoconferencing. Participants optionally provided consent for researcher access to medical records for ABI details. Demographic information, such as age, gender, ethnicity, and occupational status, were recorded. Participants then completed the baseline assessment (Time 1). AP and AE participants received a post-intervention (Time 2) and 1-month follow up (Time 3) assessment. Participants first randomised to the WL group completed a second baseline assessment (Time 2) prior to being re-randomised into either the AP or AE group.

### Secondary objective—secondary outcome measures

Detailed descriptions of secondary outcome measures are in the trial protocol [30]. Secondary outcome measures were selected by whether each was an expected outcome of BA (activities, mood, anhedonia; community integration) or a theoretically relevant process to improvements (approach and avoidance behaviours, coping with uncertainty, motivation, locus of control).

Depression and anxiety were measured using the 14-item *Hospital Anxiety and Depression Scale* (HADS [32]), with possible scores on subscales (HADS-Depression and HADS-Anxiety) ranging from 0 to 21. Example items include “I still enjoy the things that I used to” (HADS-Depression; reverse coded) and “I feel tense or ‘wound up’” (HADS-Anxiety). Higher scores indicate greater depression/anxiety.

To assess approach- and avoidance-motivated behaviours toward activities, the 20-item *Behavioural Inhibition Scale/Behavioural Activation Scale* (BIS/BAS [42]) was used. The BIS scale ranges from 7 to 28 (example item: “I worry about making mistakes), while the BAS portion comprises 3 subscales: Drive (“I go out of my way to get things I want”; range 4–16), Reward Responsiveness (“When I get something I want, I feel excited and energised”; range 5–20), and Fun Seeking (“I crave excitement and new sensations”; range 4–16). Higher scores indicate greater inhibition (BIS) or activation (BAS).

To assess difficulties with coping with uncertainty in activities, the 12-item *Intolerance of Uncertainty Scale – Short Form* (IU-SF [43]) was used. The IU-SF comprises two subscales: Prospective (“I always want to know what the future has in store for me”; range 7–35) and Inhibitory (“When it’s time to act, uncertainty paralyses me”; range 5–25). Higher scores indicate greater difficulties with either Prospective or Inhibitory uncertainty.

To assess post-traumatic stress symptoms, the 22-item *Impact of Events Scale-Revised* (IES-R [44]) was used. The IES-R has three subscales: Intrusion (“Pictures about [my injury] popped into my mind”; range 0–35), Avoidance (“I tried not to think about [my injury]”; range 0–40), and Hyperarousal (“I felt watchful and on guard”; range 0–35). A total score can also be calculated (possible range 0–110). Higher scores indicate greater post-traumatic stress.

To assess internal motivation, the 34-item *Brain Injury Rehabilitation Trust Motivation Questionnaire-Self* (BMQ-S [45]) was used. Total scores range from 34 to 136, with an example item including “I avoid doing things I don’t have to”. Higher scores indicate greater difficulties with internal motivation (i.e., not motivated).

To assess motivation for rehabilitation-related activities, the 31-item *Motivation for Traumatic Brain Injury Rehabilitation Questionnaire* (MOT-Q [46]) was used. The MOT-Q has four subscales: Lack of Denial (“I don’t have any problems worth mentioning”; range − 16 to + 16), Interest in Rehabilitation (“Rehabilitation is very useful”; range − 14 to + 14); Lack of Anger (“Therapists would have me do things that are irrelevant” [reverse coded]; range − 20 to + 20) and Reliance on Professional Help (“I rely on doctors to help me with my problems”; range − 12 to + 12). Higher scores in each subscale indicate greater motivation.

To assess community integration, the *Modified Outcome Measure – Participation Objective, Participation Subjective* (MOM-POPS [47]) was used. This is a shortened version of the POPS scale. Participants rate actual engagement in household, occupational, and social activities in the past week (Participation Objective score) and whether actual engagement differs from their ideal level of engagement in these activities (Participation Subjective score). Participants additionally list types of activities engaged in the past week (e.g., cleaned the house, made social arrangements).

To assess hedonic capacity, the 14-item *Snaith-Hamilton Pleasure Scale* (SHAPS [48]) was used. Total scores range from 0 to 14, with an example item including “*I would be able to enjoy a beautiful landscape or view.”* Higher scores indicate greater hedonic capacity.

To assess locus of control, the 12-item *Sense of Control Scale* (SCS [49, 50]) was used. The SCS comprises two subscales: Personal Mastery (“I can do just about anything I really set my mind to”; range 8–56) and Perceived Constraints (“There are many things that interfere with what I want to do”; range 4–28). Higher scores indicate greater perceived mastery or constraints, respectively.

### Additional baseline measures

The *Verbal and Spatial Reasoning Test* (VESPAR [51]) was conducted to assess cognitive function at Time 1 only. The VESPAR is a neuropsychological test of inductive reasoning using word- and picture-based subtests. Here, we used the Verbal Odd One and Spatial Odd One subtests.

At baseline only, participant expectations and credibility of their allocated group was assessed using the 6-item *Credibility/Expectancy Questionnaire* (CEQ [52]). The CEQ comprises two factors: Expectancy (“What percent improvement do you think will occur?”) and Credibility (“How confident would you be in recommending this group to a friend?”).

At Time 3, participants completed a custom-designed *Post-Study Questionnaire* (PSQ) on group experiences and factors affecting participation (see Additional File 2: Document S2).

### Exit interview

Twenty participants completed an exit interview at Time 3, comprising in-depth questions about group experiences. AP participants additionally provided feedback on content and materials (see Additional File 2: Document S3 for interview script)*.*

### Participant remuneration

Individuals taking part in-person received travel reimbursement. All participants received a £50 remuneration at Time 3.

### Data management

Measures with < 20% of missing items had total scores imputed based on averaged responses from answered items (occurred in < 5% of cases). Any measure with > 25% missing items was treated as a missing value for a participant (< 5% of cases).

WL Time 2 assessments served two purposes. These were used for comparison with Time 2 AP and AE data and also used as pre-group attendance baseline scores (i.e., effectively “Time 1”) to examine any changes post-AP or -AE group in former WL participants.

### Quantitative and qualitative data analysis

Quantitative analyses were conducted on feasibility, acceptability, and potential efficacy measures across AP, AE, and WL groups in order to determine whether trial outcomes differed as a result of initial condition allocation (e.g., greater attrition in WL participants, greater AP or AE changes in activity levels compared to no intervention).

Percent attrition rates, at what point attrition occurred, number of sessions attended (mean and median estimates in the event of extreme values), and group helpfulness and enjoyableness ratings, were calculated to inform feasibility and acceptability conclusions. Missing data and reasons for drop-out and non-attendance are reported.

Quantitative data were analysed using R statistical software version 4.1.2 [53] using an intention-to-treat analysis. Demographic variables per group are reported. Unadjusted mean differences from baseline to post-intervention between AP, AE, and WL arms with 95% confidence intervals were estimated. The Minimal Clinically Important Difference (MCID; see *Results* for details on calculation) of the BADS and secondary efficacy outcomes were estimated. A linear mixed-effects model using the BADS across time points was conducted. Exploratory analyses were conducted to estimate the effectiveness of the AP and AE groups and determine effect size estimates for a definitive trial power analysis. All data and associated code are available on the Open Science Framework database: https://osf.io/e5btr.

Qualitative data were transcribed verbatim by the first author (AK1) and research assistants (EC1, AK2, WW), with personally identifiable information removed. Qualitative data were analysed using an interpretive description thematic summary framework [54], analysed by AK1. Interpretive description aims to build clinically relevant themes and was thus deemed an appropriate method for the trial. Transcripts were analysed in sequence to build an initial codebook of potential themes that were later refined following further analysis. Given the focus on acceptability of the groups, greater description via a larger number of smaller themes was preferred (versus a more interpretive analysis of overarching themes).

As all participants received the PSQ, and 30% of participants received the exit interview, pre-specifying saturation was not possible. Potential themes relevant to acceptability were discussed in peer debriefing sessions among the supervisory team and with patient representatives using select transcripts of participants reporting positive and negative group experiences. Constructed themes were considered in light of the reflexivity entries completed by research assistants conducting the interviews and were informed by quantitative self-reported helpfulness and enjoyableness ratings of participants. Pseudonyms are used to present the data.

### Harms

Management and reporting of adverse events were conducted as per MRC protocol. Any cases of adverse events or harms are reported, alongside consideration of any potential harms due to the interventions.

## Results

**Fig. 1:**
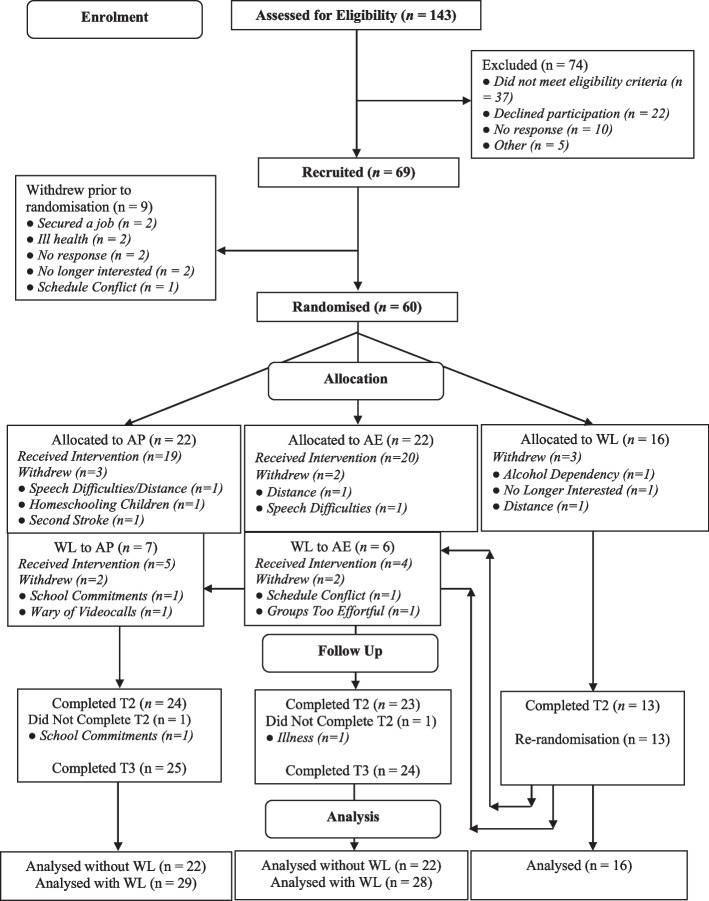
CONSORT flow chart of recruitment into the MAPLES study. AP = Activity Planning group, AE = Activity Engagement group, WL = Waitlist Controls

Sixty participants were randomised (Fig. 1), per our protocol target. Twenty-five participants were randomised pre-COVID (recruitment period March 2019–March 2020) and 35 during COVID (June 2020–January 2021). Due to the re-randomisation design, for the last recruitment wave (Wave 7, February to March 2021), participants first allocated to WL (*n* = 4) based on the predetermined randomisation sequence were immediately re-randomised to either AP or AE by opening the second randomisation envelope. This was done to ensure all participants could receive an intervention. This resulted in 22 participants each in AP (re-randomised *n* = 29), AE (re-randomised *n* = 28), and 16 WL.

AP participants began their allocated intervention at an average of 21.07 days (SD = 12.55; median = 16 days) following their baseline assessment, compared to 17.64 days (SD = 8.89, median = 15 days) for AE participants. There were 3 participants (*n* = 2 AP, *n* = 1 AE) for whom circumstances dictated significantly longer delays been initial assessment and group participation. Their delays were 119, 166, and 140 days, respectively, and are not included in the mean values to avoid potentially giving a misleading impression of the majority pattern.

The main reason for exclusion was not meeting study eligibility criteria (*n* = 37 of 74; see Additional File 2*: *Table S1 for detailed reasons for exclusion).

Participant demographics are presented in Table 2.

**Table 2:** MAPLES participant characteristics

	**Activity Planning** (*n* = 22)	**Activity Engagement** (*n* = 22)	**Waitlist Controls** (*n* = 16)
Age – M (*SD*)	52.45 (11.03)	53.59 (12.41)	54.94 (11.89)
Gender (*n*, %)			
Male	12 (54.5%)	12 (54.5%)	7 (43.8%)
Female	10 (45.5%)	10 (45.5%)	9 (56.2%)
Ethnicity (*n*, %)			
White British	21 (95.5%)	18 (81.8%)	16 (100%)
White other		2 (9.1%)	
Black British		2 (9.1%)	
Asian British	1 (4.50%)		
Type of ABI (*n*, %)			
Traumatic brain injury^a^	9 (40.9%)	6 (27.3%)	6 (37.4%)
Stroke	8 (36.4%)	13 (59.1%)	5 (31.3%)
Other	5 (22.7%)	3 (13.6%)	5 (31.3%)
Years post-ABI – M (*SD*)	7.92 (9.44)	5.91 (7.48)	7.93 (7.28)
Employment status (*n*, %)			
Full time	3 (13.6%)		
Part time	1 (4.5%)	3 (13.6%)	2 (12.5%)
Unemployed	7 (31.8%)	5 (22.7%)	2 (12.5%)
Medical retirement	6 (27.3%)	9 (40.9%)	5 (31.2%)
Retirement	3 (13.6%)	3 (13.6%)	2 (12.5%)
On leave		2 (9.10%)	4 (25.0%)
Student	1 (4.5%)		1 (6.0%)
Psychotropic medication (*n*, %)			
Antidepressant	8 (36.3%)	8 (36.3%)	5 (31.2%)
Antianxiety	3 (13.6%)	5 (22.6%)	3 (18.8%)
Current clinical input^b^ (*n*, %)			
None	17 (77.3%)	17 (77.3%)	14 (87.5%)
Clinical psychology	3 (13.7%)	3 (9.1%)	1 (6.2%)
Occupational therapy	1 (4.5%)	2 (9.1%)	
Physiotherapy	1 (4.5%)		
Other			1 (6.2%)
Inductive reasoning – M (*SD*)			
Verbal	101.56 (10.46)	102.48 (6.67)	109.48 (8.81)
Spatial	103.31 (5.02)	103.57 (6.33)	105.59 (7.39)

*ABI* Acquired brain injury

^a^Across the three trial arms, this included 7 out of 21 individuals with mild TBI

^b^Participants were recorded as having “current clinical input” even if this was very occasional contact or being on a therapists’ client list with no active treatment over the study period

### Primary objective—feasibility outcomes

Due to COVID-19 requiring completely remote research, feasibility data were summarised for in-person and remote sessions separately.

### Recruitment outcomes

Recruitment targets were met only when including re-randomised WL participants; otherwise, minimum recruitment targets were only met for Wave 1 and Wave 4 (Table 3).

**Table 3:** Total number of new participants recruited per wave, with participants re-randomised to an active intervention (Activity Planning or Activity Engagement) included in brackets. Recruitment for solely online adaptations of the active interventions began in Wave 4

Protocol target	Actual						
Minimum 9, Maximum 18 participants recruited per wave	Wave 1 Jun–Jul 2019	Wave 2 Sep–Nov 2019	Wave 3 Feb–Apr 2020	Wave 4 Jun–Jul 2020	Wave 5 Sep–Nov 2020	Wave 6 Oct–Dec 2020	Wave 7 Feb–Mar 2021
	9	8 (+ 3 WL)	8 (+ 1 WL)	11 (+ 2 WL)	8 (+ 4 WL)	7 (+ 0 WL)	8 (+ 2 WL)

*WL* Waitlist Controls

Before the first UK COVID-19 lockdown (March 23 2020), the majority of participants recruited were from NHS ABI services (19 of 25). During COVID-19, participants were mostly recruited from ABI charities (22 of 35; see Additional File 2*: *Table S2 for eligibility per recruitment source). Self-referral via social media was the most efficient referral route (14 of 21 screened randomised; 66.7% eligible).

### Study withdrawal

When including re-randomised participants, attrition was less than 20% across the three arms (Table 4). Not including those re-randomised, withdrawal rates were 13.6%, 9.1%, and 18.8% in AP, AE, and WL, respectively.

**Table 4:** Summary of attrition between the Activity Planning and Activity Engagement groups

Protocol target	Actual			
Attrition less than 20% across all trial arms		Activity Planning group (*n* = 29)	Activity Engagement group (*n* = 28)	Waitlist Controls (*n* = 16)
	In-person	*n* = 2	*n* = 1	*n* = 2
	Online	*n* = 3	*n* = 3	*n* = 1
	Total	*n* = 5 (17.2%)	*n* = 4 (14.8%)	*n* = 3 (18.8%)

The most common reason for withdrawal was difficulties travelling to the study location (*n* = 3; 1 per trial arm), followed by expressive aphasia affecting group participation (*n* = 1 AP and *n* = 1 AE) (see Fig. 1 for all reasons).

### Intervention session attendance

Including participants who withdrew, average attendance did not differ between the AP and AE group (*t* =  − 0.38, *p* = 0.70; Table 5). However, median attendance rose from 7 to 8 when delivered online. Average attendance did not differ in-person or online for the AP (*t* =  − 1.25, *p* = 0.22) or AE group (*t* =  − 0.55, *p* = 0.59). Session attendance per wave and reasons for non-attendance are in Additional File 2*: *Figures S1 and S2*.*

**Table 5:** Summary of average attendance between the Activity Planning and Activity Engagement groups, including those who withdrew

Protocol target	Actual		
At least 5 out of 8 sessions attended		Activity Planning group	Activity Engagement group
	In-person	Mean = 5.90 (SD = 2.33) Median = 7	Mean = 6.50 (SD = 1.18) Median = 7
	Online	Mean = 6.89 (SD = 1.81) Median = 8	Mean = 6.88 (SD = 1.93) Median = 8

### Acceptability outcomes

#### Credibility and expectations of interventions

Baseline CEQ summary data are in Additional File 2*: Table S3*. In terms of how logical each group was perceived to be, the AP group had a mean of 7.24 (SD = 1.62) compared to the AE group (*M* = 6.61, SD = 2.33). Comparable means were found for perceptions of how effective each group would be in increasing activity levels (AP *M* = 7.00 [SD = 1.73]; AE *M* = 6.32 [SD = 2.45]), in recommending the group to a friend (AP *M* = 6.86 [SD = 1.77]; AE *M* = 6.68 [SD = 2.39]), and in reporting feeling that their activity levels would improve (AP *M* = 6.45 [SD = 2.13]; AE *M* = 5.89 [SD = 2.36]).

#### Post-study questionnaire—quantitative data

PSQ responses were positive for both groups (Table 6). Irrespective of mode of delivery, AP and AE were rated as similarly enjoyable (*t* = 0.27, *p* = 0.79) and helpful (*t* = 1.99, *p* = 0.05). The AP group was rated as similarly helpful (*t* = 0.43, *p* = 0.67) and enjoyable (*t* = 0.14, *p* = 0.89), whether in-person or online. The AE group was similarly helpful online and in-person (*t* =  − 0.56, *p* = 0.58), though rated as more enjoyable online (*t* =  − 2.54, *p* = 0.02).

**Table 6:** Summary of responses to Post-Study Questionnaire (PSQ) per group for participants who attended in-person versus online

PSQ item	Activity Planning		Activity Engagement	
	In-person	Online	In-person	Online
Helpfulness of group –				
*M* (SD)				
Min–max	8.50 (1.07)	8.24 (1.56)	7.00 (2.40)	7.50 (1.99)
Median	7–10 8	5–10 8	3–10 7.5	2–10 8
Enjoyableness of group –				
*M* (SD)				
Min–max	8.75 (1.58)	8.65 (1.73)	7.50 (2.37)	9.29 (0.99)
Median	6–10 9.5	4–10 9	3–10 8	7–10 10
Number of barriers to attendance –				
M (SD)				
Min–max	1.75 (1.67)	3.35 (2.23)	1.20 (1.40)	2.64 (1.82)
Median	0–4 2	0–8 3	0–4 1	0–7 3

#### Barriers to attendance

**Fig. 2:**
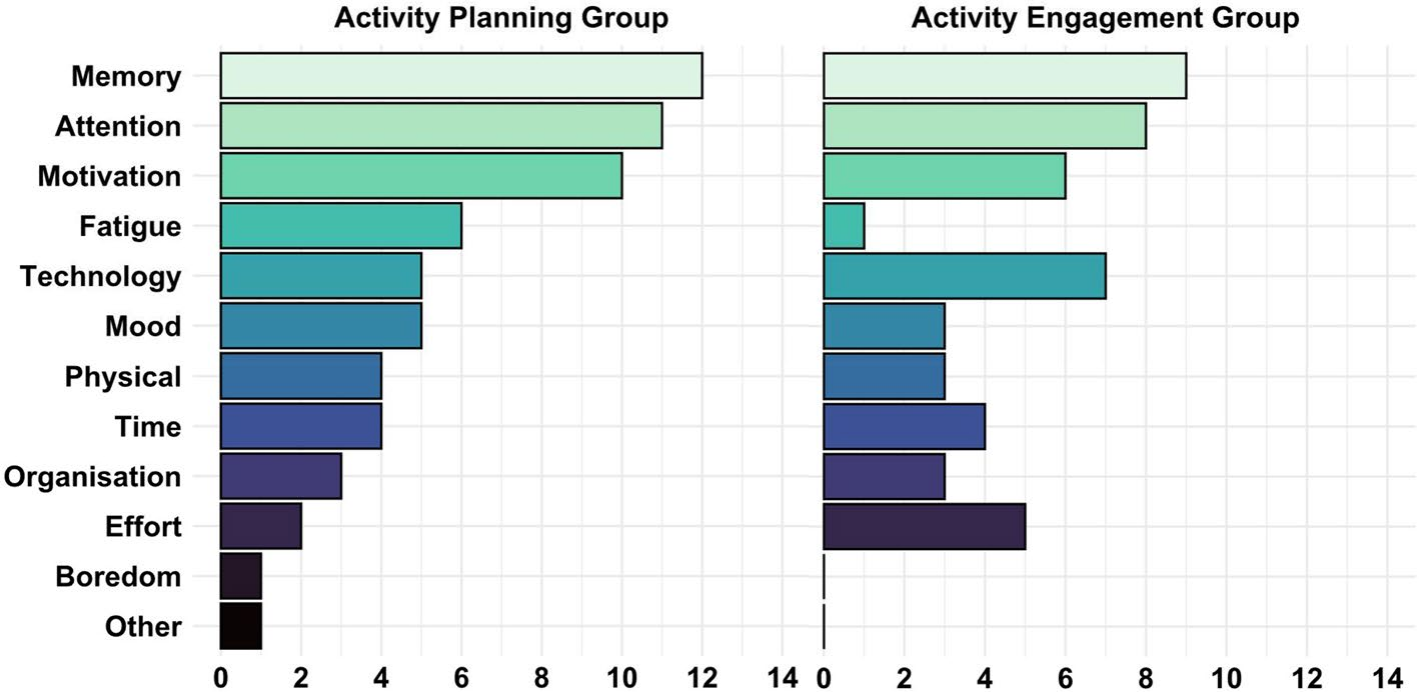
Frequency of each barrier to attendance across all participants in the AP group (left) and AE group (right)

All participants (including those who withdrew) were asked whether there were any barriers to attending study sessions, even if they were able to overcome these barriers. Average number of barriers to attending the AP (M = 2.84) and AE (M = 2.04) groups did not significantly differ (*t* = 1.40, *p* = 0.17). There appeared to be variations in barriers present (Fig. 2), though only fatigue being less frequently reported as a barrier in AE was statistically significant (*t* = 2.99, *p* < 0.01).

The number of barriers in the AP group was not statistically different online compared to in-person (*t* =  − 1.79, *p* = 0.09). There were no statistically significant differences in number of barriers for AE online versus in-person (*t* =  − 2.10, *p* = 0.05). Regardless of group, attending online resulted in more reported attention, fatigue, technology and organisational barriers (*t*s =  − 2.89 to − 3.78, *p*s < 0.01). For AP specifically, technology, fatigue, and attention barriers were more frequently reported online (*t*s =  − 2.58 to − 4.24, *p*s < 0.05). For AE specifically, organisational barriers were more frequently reported online (*t* =  − 2.28, *p* = 0.04).

### Acceptability outcomes—qualitative feedback on groups

PSQ and exit interview qualitative data were used to explore perspectives of acceptability, specifically aspects that positively or negatively contributed to helpfulness or enjoyableness, of the groups and overall participation in the study. Table 7 synthesises perceived strengths of the groups or areas for improvement, with example quotes in Additional File 2*: *Table S4. Recommendations presented below should be considered within any local context of recreating the groups described here. For space, in-depth qualitative data analysis of specific group experiences will be reported elsewhere.

**Table 7:** Recommendations for important areas to maintain or modifications of the groups, based on qualitative data

	**Activity Planning group**	**Activity Engagement group**	**Both groups**
Group factors	Perceived benefits and prerequisites for group success		
	• Sharing group materials ahead of each session • Providing a summary of session content following session end	• Having a variety of activities suitable for different types of ABI • Group activities were mood-enhancing	• Learning from other group members • Day-before and day-of reminders of sessions
	Suggestions for improvement		
	• Paced information • Time limits for group discussion relative to session content	• Prioritise group discussion • More discussion of ABI	• Dedicated time for group members to interact without facilitator • Adaptable materials for different issues for different ABIs
Study factors	Perceived benefits and prerequisites for group success		
	• Wide range of relevant strategies • Learning from other members’ activities	• Sharing personal interests with others	• Dedicated transport support/reimbursement for in-person sessions • Regular breaks • Remote delivery
	Suggestions for improvement		
	• Incorporate more explicit session material on sharing ABI story • Reduce content per session, or • Increase session duration	• Increase session duration • Clearer rationale for potential benefits of group • Explicit discussion about mood	• Increase minimum group size • Enhance group cohesion across variety of member characteristics • Dedicated tech support for online sessions • Pre-select participants with similar characteristics/ issues
Facilitator factors	Perceived benefits and prerequisites for group success		
	• Sufficient training delivering session materials • Empathic response when activities were not completed	• Regularly offering choice in selecting activities • Using low-demand activities that just had a few steps to learn	• Acknowledging individuality of ABI effects • Engaging but not forceful facilitation • Being available if needed between sessions
	Suggestions for improvement		
	• Communicate session topics using clear language • Keeping discussions suitably focused	• Group protocol should specify need to adapt sessions based on participant needs • Fatigue management for in-session activities	• Ensure equal participation from group members

#### Supporting participants with aphasia

To evaluate the potential suitability of the groups for people with language difficulties, qualitative data from participants with aphasia are presented in Additional File 2: Document S4 and Table S5*.* In brief, participants with fluent aphasia seemed to have positive experiences within groups; however, for those with non-fluent aphasia, individual sessions were preferred.

### Secondary objective—clinical outcomes

The secondary objective was to provide estimates on the primary efficacy outcome measure and sample size determination for a subsequent trial. We summarise missing data, AP and AE fidelity assessment results, and efficacy estimations on the primary efficacy outcome measure between groups.

#### Fidelity assessment results

Both the AP and the AE groups were delivered as intended, with percent fidelity estimates of 95.06% (SD = 6.59, range 80–100%) and 99.17% (SD = 2.89, range 90–100%), respectively.

#### Missing data and acceptability of questionnaires

Generally, participants who did not withdraw had complete data.

Reasons for non-completion included discomfort with the measure content (*n* = 1 SHAPS; AE group), providing opinions about rehabilitation staff (*n* = 1 MOT-Q; AE group), due to technical errors with online data collection (*n* = 1 BMQ-S AP group; *n* = 1 MOM-POPS AP group) or accidental omission from the questionnaire battery (*n* = 1 MOT-Q WL group), or for unknown reasons (*n* = 1 MOT-Q WL group; *n* = 1 SCS AE group).

#### Baseline outcome measures

Randomisation produced well-matched groups on study variables (see Table 8).

**Table 8:** Scores on all outcome measures at baseline by group

	**Activity Planning** (*n* = 22)	**Activity Engagement*** (n* = 22)	**Waitlist Control** (*n* = 16)
BADS			
Total	94.3 (27.2)	80.6 (31.4)	93.4 (20.4)
Activation	22.4 (8.4)	19.2 (8.9)	19.0 (8.6)
Lack of avoidance/rumination	31.5 (11.9)	26.6 (12.4)	30.1 (10.0)
Lack of school/work impairment	18.6 (6.3)	16.6 (8.1)	20.4 (5.7)
Lack of social impairment	21.7 (8.7)	18.3 (8.1)	23.9 (4.8)
HADS			
Depression	9.1 (5.2)	11.5 (4.3)	8.3 (4.8)
Anxiety	8.1 (5.4)	9.1 (5.9)	7.8 (5.3)
BMQ-S	83.5 (13.9)	87.9 (16.2)	81.4 (15.8)
MOT-Q			
Lack of denial	6.9 (5.9)	10.2 (4.9)	6.1 (6.0)
Interest in rehabilitation	8.1 (5.2)	8.5 (4.0)	5.9 (5.9)
Lack of anger	8.9 (6.0)	9.1 (5.8)	8.3 (6.9)
Reliance on professional help	6.1 (3.5)	4.2 (3.5)	3.4 (3.9)
IES-R			
Total	27.2 (14.5)	33.3 (23.4)	25.6 (15.2)
Avoidance	10.8 (5.5)	11.9 (8.01)	7.8 (4.6)
Hyperarousal	7.5 (5.2)	9.3 (6.9)	7.4 (6.0)
Intrusions	9.55 (6.61)	12.1 (9.47)	10.5 (5.9)
BIS/BAS			
Inhibition	20.4 (4.1)	20.6 (5.1)	21.4 (4.6)
Fun seeking	10.0 (2.4)	9.2 (2.5)	9.2 (2.6)
Reward responsiveness	16.5 (3.7)	15.4 (3.0)	15.9 (3.2)
Drive	10.1 (3.6)	10.5 (2.8)	10.3 (3.2)
SCS			
Perceived mastery	20.3 (3.1)	18.3 (5.4)	20.1 (5.6)
Perceived constraints	32.6 (10.9)	36.8 (10.3)	34.0 (9.0)
MOM-POPS			
Number of weekly activities	10.8 (4.8)	10.3 (4.4)	10.4 (3.9)
SHAPS	12.3 (2.9)	11.5 (2.3)	12.4 (2.3)
IU-SF			
Prospective anxiety	21.5 (6.4)	21.9 (6.2)	20.8 (7.3)
Inhibitory anxiety	14.8 (5.0)	16.4 (4.3)	13.1 (4.7)

*BADS* Behavioural Activation for Depression Scale, *HADS* Hospital Anxiety and Depression Scale, *BMQ-S* BIRT Motivation Questionnaire-Self, *MOT-Q* Motivation for Traumatic Brain Injury Rehabilitation Questionnaire, *IES-R* Impact of Event Scale-Revised, *BIS/BAS* Behavioural Inhibition/Behavioural Activation Scales, *SCS* Sense of Control Scale, *MOM-POPS* Modified Outcome Measure-Participation Objective, Participation Subjective, *SHAPS* Snaith-Hamilton Pleasure Scale, *IU-SF* Intolerance of Uncertainty Scale-Short Form

#### Efficacy of intervention on primary efficacy outcome measure

The unadjusted mean difference on the BADS from Time 1 to Time 2 was − 10.41 (95% CI − 19.67 to − 1.27) for the AP group, compared to − 7.35 (95% CI − 14.84 to 0.14) in the AE group and − 1.51 (95% CI − 13.78 to 10.76) for the WL group, indicating positive trend toward improvements in activity levels in the AP and AE groups.

A mixed-effects linear model was conducted using total BADS scores via the *lmerTest* R package [55]. Participants were initially modelled as a random effect. However, adding BADS baseline scores as a covariate resulted in model singularity. As participants were recruited in “waves” where the AP, AE, and WL groups ran in parallel, we instead considered cohort effects because (a) this allowed accounting for COVID-19 onset and resultant fluctuating restrictions on socialising, mood, and activity levels; (b) COVID-19 dramatically affected recruitment, and (c) in the last wave, those first sent to WL were immediately re-randomised. Whilst randomisation should protect against potential systematic differences, study wave (Waves 1–7) was modelled as a random effect to account for this.

Missing data was estimated using restricted maximum likelihood estimation. F-statistics with effective degrees of freedom were estimated using Satterthwaite’s method in *lmerTest*. For each model, adjusted intraclass correlation coefficients were used to estimate the amount of variance attributable to the random effects [56]. Tukey-adjusted post-hoc tests were conducted using estimated marginal means. Full mixed-effects model results (including b-values and standard errors) are shown in Additional File 2*: *Table S6*.*

**Fig. 3:**
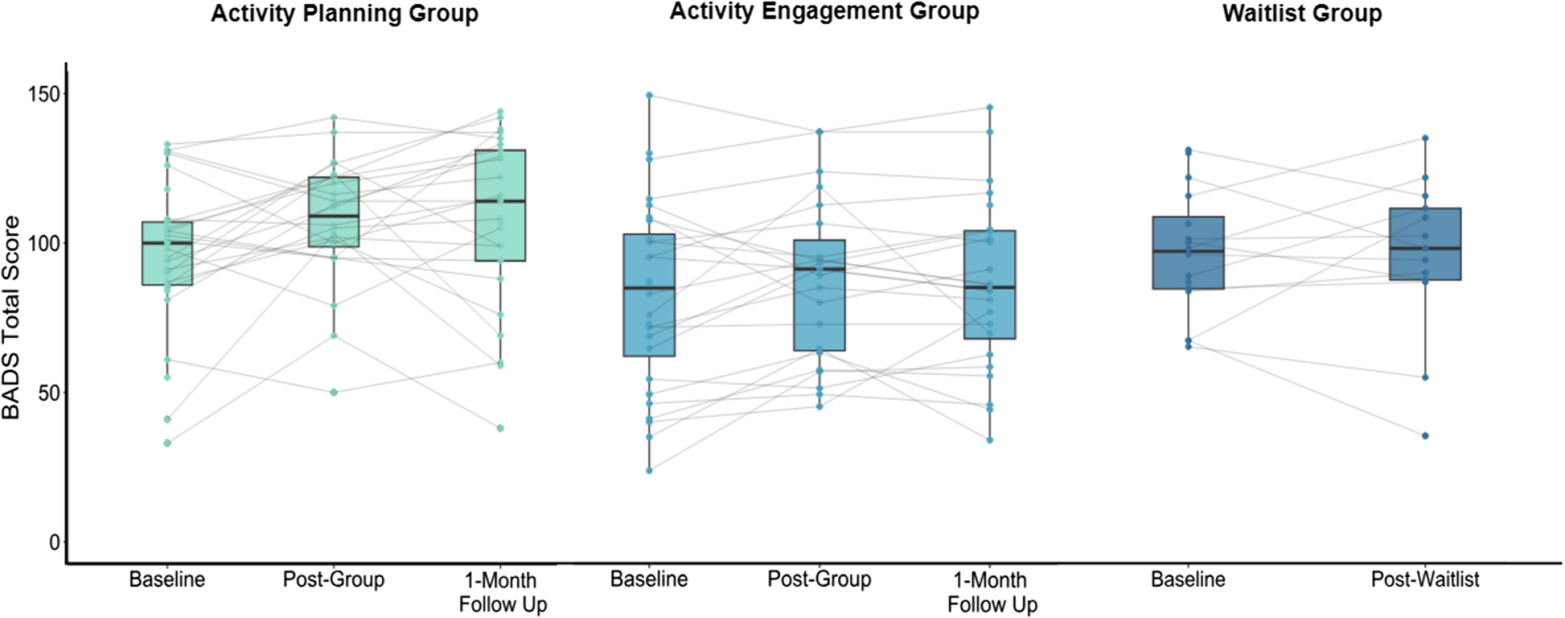
Visualisation of individual change between the three groups on the Behavioural Activation for Depression Scale (BADS) across each time point. Visualisations include those who were re-randomised into either the AP or AE group. Higher BADS scores represent greater activity engagement (i.e., improvement)

On the BADS (Fig. 3), there was a main effect of Time (Satterthwaite’s *F*_2,167_ = 3.82, *p* < 0.05) and Group (Satterthwaite’s *F*_2,170_ = 3.33, *p* < 0.05) and baseline scores (Satterthwaite’s *F*_2,172_ = 479.43, *p* < 0.001), but no Time by Group interaction (Satterthwaite’s *F*_3,167_ = 0.98, *p* = 0.39). Random effects estimates were low (14.44, *SD* = 3.80, χ^2^ = 5.54, *p* = 0.02, ICC_adj_ = 0.07), indicating that variation in participant intercept due to study wave was likely minor. In post-hoc tests, only AP participants demonstrated improvements on BADS scores from Time 1 to Time 2 (*t* =  − 2.76, *p* = 0.01) and to Time 3 (*t* =  − 2.68, *p* = 0.02). Time 2 and Time 3 scores did not differ within the AP group (*t* = 0.10, *p* = 0.99). BADS scores in the AE group did not differ between Time 1 to Time 2 (*t* =  − 1.76, *p* = 0.18) or to Time 3 (*t* =  − 1.12, *p* = 0.50). WL participants showed no BADS improvements from Time 1 to Time 2 (*t* =  − 0.18, *p* = 0.98). In summary, significant gains in BADS scores were only observed in participants randomised to the AP group and these improvements were well maintained at Time 3.

#### Exploratory analyses—secondary efficacy outcome measures

To descriptively examine potential effects between groups across study measures, a summary of mean differences with 95% CIs across all study outcome measures is shown in Table 9. A visualisation of point estimates is in Additional File 2*: *Figure S3*.*

**Table 9:** Unadjusted mean differences on study outcome measures from baseline to post-intervention

**Mean difference (95% CI)**	**Activity Planning** (*n* = 22)	**Activity Engagement** *(n* = 22)	**Waitlist Control** (*n* = 16)
**BADS**			
Total	− 10.42 (− 19.67 to − 1.17)	− 7.35 (− 14.84 to 0.14)	− 1.51 (− 13.78 to 10.76)
**HADS**			
Depression	1.39 (− 0.08 to − 2.86)	2.22 (0.81 to 3.63)	− 1.95 (− 3.63 to − 0.27)
Anxiety	2.21 (0.93 to − 3.49)	− 0.30 (− 1.24 to 0.63)	− 1.17 (− 3.98 to 1.65)
**BMQ-S**	7.19 (2.26 to 12.13)	2.04 (− 1.03 to 5.11)	− 2.17 (− 6.24 to 1.89)
**MOT-Q**			
Lack of denial	0.44 (− 1.22 to 2.11)	1.48 (− 0.28 to 3.24)	− 0.05 (− 3.46 to 3.36)
Interest in rehabilitation	0.17 (− 1.77 to 2.12)	0.39 (− 2.35 to 3.12)	− 2.08 (− 4.31 to 0.16)
Lack of anger	− 2.13 (− 3.91 to − 0.35)	− 0.18 (− 2.54 to 2.17)	0.00 (− 2.60 to 2.60)
**IES-R**			
Avoidance	1.47 (− 0.54 to 3.47)	− 0.13 (− 2.32 to 2.06)	1.68 (0.04 to 3.31)
Hyperarousal	1.96 (0.70 to 3.21)	0.85 (− 1.62 to 3.32)	0.58 (− 2.73 to 3.89)
Intrusions	2.58 (0.62 to 4.54)	− 1.09 (− 3.43 to 1.26)	2.86 (− 0.65 to 6.36)
**BIS/BAS**			
Inhibition	0.51 (− 1.07 to 2.09)	0.50 (− 0.85 to 1.85)	− 0.56 (− 2.94 to 1.82)
Reward responsiveness	− 0.54 (− 1.93 to 0.85)	− 1.22 (− 2.69 to 0.26)	− 0.18 (− 1.77 to 1.42)
Drive	0.21 (− 0.88 to 1.29)	− 0.58 (− 1.59 to 0.45)	− 0.06 (− 1.89 to 1.78)
**SCS**			
Perceived mastery	− 0.63 (− 2.37 to 1.12)	− 1.26 (− 3.47 to 0.95)	2.18 (− 2.03 to 6.39)
Perceived constraints	2.04 (− 2.09 to 6.17)	0.17 (− 2.04 to 2.39)	0.31 (− 3.95 to 4.57)
**SHAPS**	− 0.34 (− 0.90 to 0.22)	− 0.56 (− 1.74 to 0.61)	− 0.09 (− 1.48 to 1.29)
**IU-SF**			
Prospective anxiety	0.04 (− 2.03 to − 2.11)	− 2.30 (− 4.93 to 0.32)	1.46 (− 2.08 to 5.01)
Inhibitory anxiety	2.91 (1.13 to 4.68)	0.74 (− 1.34 to 2.81)	− 1.71 (− 4.98 to 1.56)

Of note, negative scores on the BADS, all MOT-Q subscales, BAS-Reward Responsiveness, BAS-Drive, SCS-Perceived Mastery, and SHAPS indicate improvement. Positive scores on the HADS subscales, BMQ-S, IES-R subscales, BIS Inhibition, SCS-Perceived Constraints, and IU-SF subscales indicate improvement

*BADS* Behavioural Activation for Depression Scale, *HADS* Hospital Anxiety and Depression Scale, *BMQ-S* BIRT Motivation Questionnaire-Self, *MOT-Q* Motivation for Traumatic Brain Injury Rehabilitation Questionnaire, *IES-R* Impact of Event Scale-Revised, *BIS/BAS* Behavioural Inhibition/Behavioural Activation Scales, *SCS* Sense of Control Scale, *SHAPS* Snaith-Hamilton Pleasure Scale, *IU-SF* Intolerance of Uncertainty Scale-Short Form

To examine potential statistical effects on secondary efficacy outcome measures, exploratory analyses on the HADS, BMQ-S, the SCS, and IU-SF were conducted. These were selected based on MCID results (presented below). For ease of exposition, they are reported here because the analysis method was identical to that of the BADS (above).

There was a significant time by group interaction on the HADS-Depression subscale (*F*_3,167_ = 5.96, *p* < 0.001; VC = 0.31, SD = 0.56, ICC_adj_ = 0.06). In post hoc contrasts, both AP (*t* =  − 4.30*, p* < 0.001) and AE (*t* =  − 4.60*, p* < 0.001) participants demonstrated *reduced* HADS-Depression scores versus WL at Time 2, whilst AP and AE participants did not differ at Time 2 (*t* = 0.41, *p* = 0.91) nor at Time 3 (*t* =  − 0.84, *p* = 0.68). In summary, both intervention groups were equally effective in reducing HADS-Depression scores (relative to Waitlist) and these improvements were maintained from Time 2 to Time 3 for both AP (*t* = 0.01, *p* = 1.00) and AE participants (*t* =  − 1.25, *p* = 0.43).

For HADS-Anxiety scores, there was a significant Time by Group interaction (*F*_3,167_ = 4.04, *p* < 0.01; VC = 0.15, SD = 0.38, ICC_adj_ = 0.03). At Time 2, AP participants showed significantly greater *reductions* in anxiety than both AE (*t* =  − 3.98*, p* < 0.001) and WL participants (*t* =  − 4.13*, p* < 0.001). AE did not differ from WL (*t* =  − 0.78, *p* = 0.72). HADS-Anxiety reductions within AP were maintained from Time 2 to Time 3 (*t* =  − 0.83, *p* = 0.69) and were still lower than AE (*t* =  − 2.55, *p* = 0.03). In summary, only Activity Planning group participants showed significant reductions in HADS-Anxiety scores that were well maintained at Time 3.

**Fig. 4:**
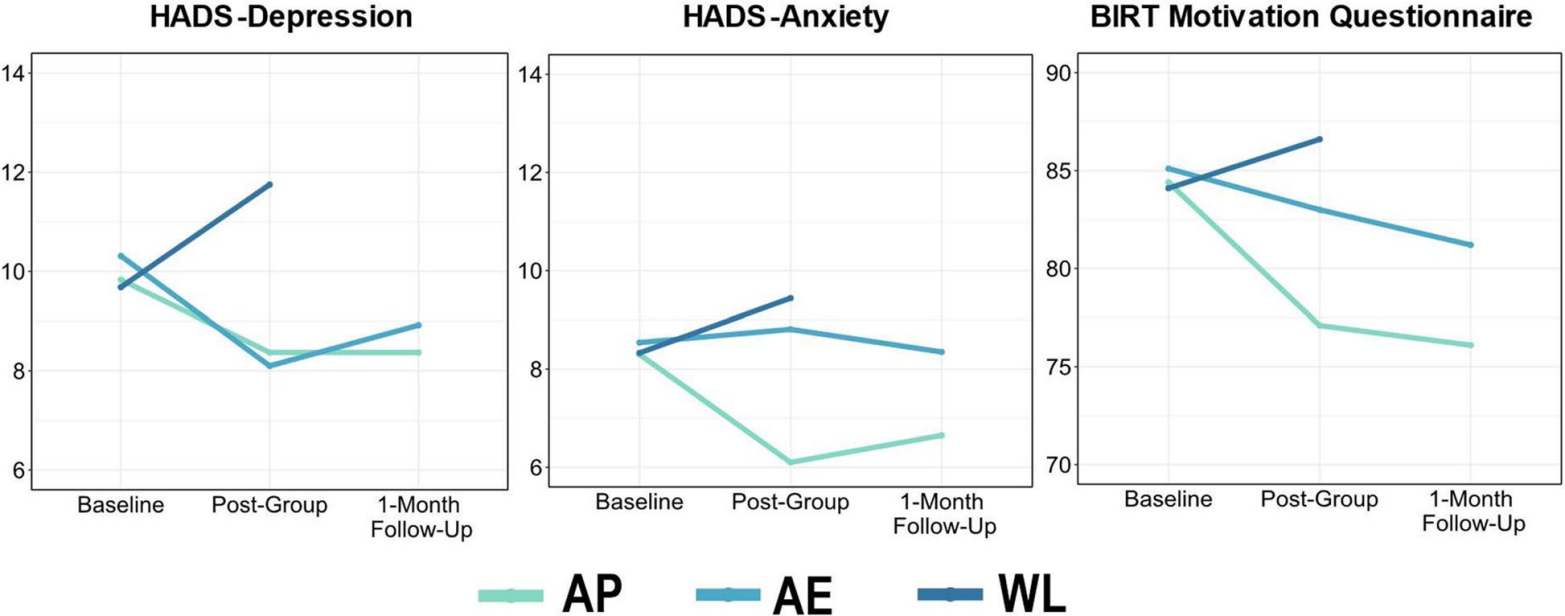
Changes in depression, anxiety, and motivation between the three groups at baseline, post-group, and 1-month follow-up. AP Activity Planning, AE Activity Engagement, WL Waitlist Controls, HADS Hospital Anxiety and Depression Scales, BMQ-S Brain Injury Rehabilitation Trust Motivation Questionnaire

There was a significant Time by Group interaction on the BMQ-S (*F*_3,164_ = 3.66, *p* < 0.05; VC = 2.98, SD = 1.73, ICC_adj_ = 0.06). Only AP participants demonstrated reductions in motivation difficulties versus AE (*t* =  − 2.91*, p* < 0.05) and WL (*t* =  − 3.99, *p* < 0.001) at Time 2 and compared to AE at Time 3 (*t* =  − 2.59, *p* < 0.05). AE and WL participants did not differ at Time 2 (*t* =  − 1.54, *p* = 0.27). BMQ-S reductions in AP were maintained from Time 2 to Time 3 (*t* = 0.49, *p* = 0.87). In summary, again only AP group participants showed significant improvements in motivation which were well maintained at time 3. Changes in HADS-Depression and Anxiety, and in BMQ-S scores, are illustrated in Fig. 4.

There were no statistically significant differences between groups on the Perceived Constraints (*p* = 0.77) and Perceived Mastery (*p* = 0.17) SCS subscales.

On the IU-SF Inhibitory subscale, there was a significant Time by Group interaction (*F*_3,167_ = 3.27, *p* = 0.02, VC = 0.27, SD = 0.53, ICC_adj_ = 0.03). At Time 2, AP participants had greater Inhibitory Anxiety reductions versus AE (*t* =  *− *2.64,* p* = 0.02) and WL (*t* =  *− *3.86, *p* < 0.001). AE and WL participants did not differ from each other (*t* =  *− *1.63, *p* = 0.24). AP participants maintained reductions from Time 2 to Time 3 (*t* =  *− *0.30, *p* = 0.95). There was no interaction on the IU-SF Prospective subscale. To summarise, again only AP participants showed significant reductions in intolerance of uncertainty Inhibitory Anxiety that were well maintained at Time 3. There were no group effects on the IU-Prospective Anxiety items.

Taken together, results suggest that participants in the AP group had a wider range of improvements in activity levels, depression and anxiety symptoms, difficulties with motivation, and the inhibiting effects of uncertainty and that these improvements persisted for at least a month post-intervention. There were also significant reductions in depression scores in the AE group, relative to Waitlist, that were well-maintained. These outcomes did not seem affected by study wave, though further modelling for random slopes in a larger trial would be beneficial.

Caution is needed in exploratory analyses due to multiple comparisons. For this reason, we analysed secondary efficacy measures based on MCID estimates (see below). In addition, the *pattern* of results is important. Where a set of conceptually related measures (anxiety, low mood, low motivation) show *consistent* patterns across the groups, the likelihood of this reflecting a true underlying pattern is increased.

### Estimation of the Minimal Clinically Important Difference (MCID)

The MCID for all outcome measures was calculated using the standard error of measurement (SEM):

$$SEM= \sigma x \sqrt{1-r}$$

Sigma ($\sigma$) was Time 1 standard deviation and the reliability (*r*) was Time 1 internal consistency. Time 1 to Time 2 data were used to calculate percent estimates across groups, including those re-randomised. For the BADS, the MCID was 8.28. Missing data at Time 2 was imputed using predictive mean matching (PMM) across 5 imputed datasets via the *mice* R package [57].

**Fig. 5:**
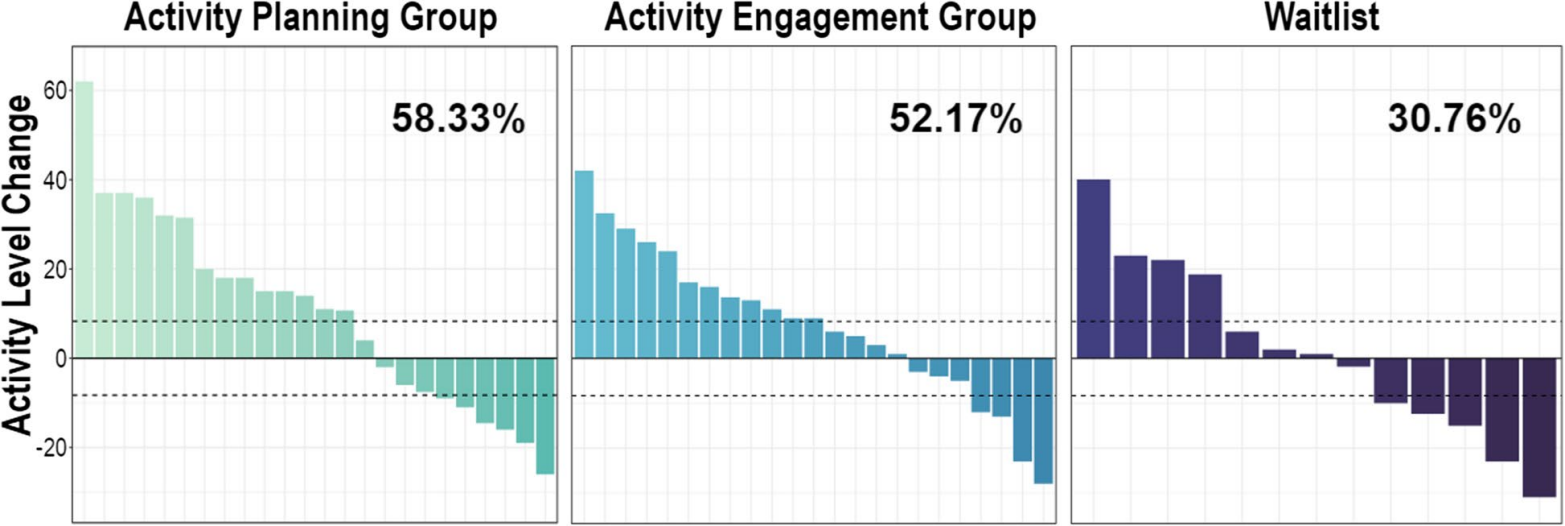
Visualisations of participant-level change scores from Time 1 to Time 2 on the Behavioural Activation for Depression Scale (BADS), not including those who withdrew. Dashed lines indicate the minimal clinically important difference (8.28) in either direction. Higher change scores indicate greater improvements in activity levels. Percentages indicate numbers of those within in each group who made MCID improvements on the BADS from Time 1 to Time 2

BADS MCID change from Time 1 to Time 2 between groups using complete cases (including those re-randomised) are in Fig. 5, where 58.33% of AP participants showed BADS improvements at or above this minimally clinically important level, versus 52.17% of AE participants and 30.77% of WL participants. Across 5 imputed datasets, AP participants had a MCID improvement range of 51.72–65.52% versus AE (range 42.86–57.14%) and WL (range 25–37.5%).

As shown in Fig. 5, there were also MCID on this primary efficacy outcome measure in the opposite direction in all groups (AP 25%; imputed range 20.69–34.48%; AE 17.39%; imputed range 17.86–32.14%; WL 38.46% imputed range 31.25–50.0%).

#### Exploratory analysis–secondary outcome measures MCID

**Fig. 6:**
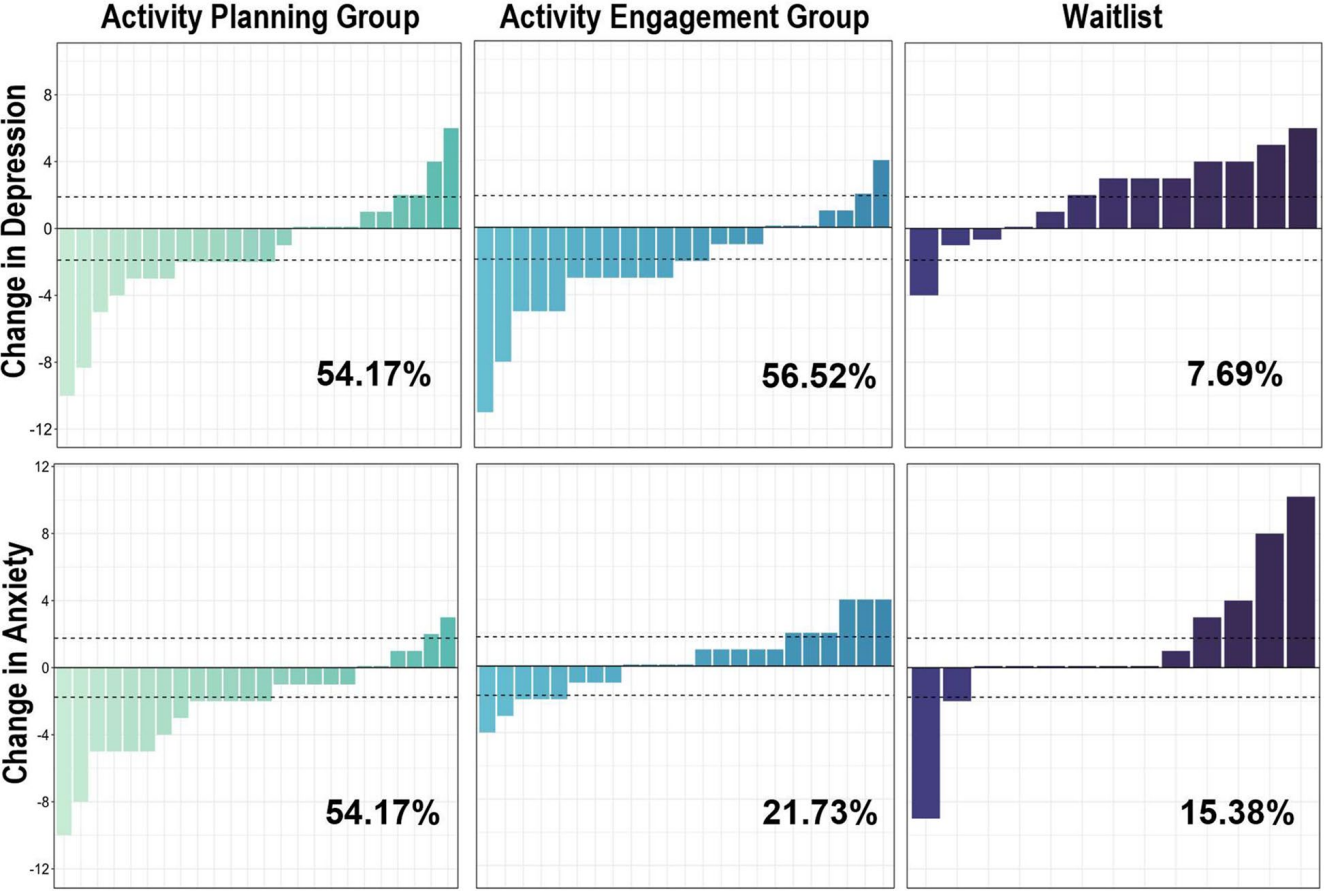
Visualisations of participant-level change scores from Time 1 to Time 2 on the Hospital Anxiety and Depression Scale (HADS), not including those who withdrew. HADS-Depression change scores are on the top row, and HADS-Anxiety scores are in the bottom row. Dashed lines indicate the minimal clinically important difference (HADS-Depression = 1.96, HADS-Anxiety = 1.83) in either direction. Lower change scores indicate greater reductions in depression and anxiety. Percentages indicate numbers of those within in each group who made MCID improvements on either measure from Time 1 to Time 2

Changes in MCID on secondary outcome measures were explored to determine which variables may be useful for a definitive trial. HADS-Depression and HADS-Anxiety MCID changes are presented in Fig. 6. This shows that 54.17% of AP participants demonstrated clinically meaningful reductions on the HADS-Depression scale (versus 56.52% in AE and 7.69% in WL). In terms of HADS-Anxiety, 54.17% of AP participants demonstrated clinically meaningful reductions compared to 20.83% AE and 15.38% WL.

Across five imputed datasets, AP participants had a MCID improvement range of 51.72–58.62%, versus AE (range 57.14–64.28%), and WL (18.75% across all five imputations) on HADS-Depression. For HADS-Anxiety, the imputed range was 51.72–55.17% for AP, versus AE (range 21.42–28.57%), and WL (range 18.75–31.25%).

Complete case MCID improvements on study measures from Time 1 to Time 2 are in Additional File 2*: *Table S7. The most responsive measures were the HADS, the BMQ-S, the SCS, and the IU-SF.

#### Sample size analysis for future trials

BADS Cohen’s *d* estimates were obtained using estimated marginal means via the *emmeans* R package [58], adjusted for baseline BADS scores. Given its relevance to BA, HADS-Depression effect sizes were also estimated.

At Time 2, BADS effect size for AP vs AE was 0.48 (95% CI =  − 0.12 to 1.09) and 0.79 (95% CI 0.08 to 1.50) against WL. The effect size for AE versus WL was 0.31 (95% CI =  − 0.40 to 1.02).

At Time 2, HADS-Depression effect sizes for AP versus AE were 0.18 (95% CI =  − 0.42 to 0.79) and − 1.63 (95% CI − 2.36 to − 0.89) against WL. The effect size for AE versus WL was − 1.82 (95% CI =  − 2.56 to − 1.07).

Power analyses were conducted using raw BADS and HADS-D estimates, and a range of estimates (range 0.30 to 1.00, based on the Oates et al. [15] systematic review) to account for likely effect size fluctuations in future trials. Power analyses (alpha = 0.05, beta = 0.80) were two-tailed and were conducted for all possible primary comparisons of interest (e.g., detecting effects between only AP and AE). Depending on the primary comparison of interest and outcome measure used, estimates varied widely (Table 10). For example, detecting a difference in BADS scores between AP and WL groups would require 26 participants per group based on raw estimates. Similarly, detecting a difference in HADS-D scores between either AP or AE to WL would require as little as 6 participants per group based on raw estimates; however, detecting a difference in HADS-D scores between AP and AE groups would require 485 participants per group.

**Table 10:** Sample size calculations based on effect size estimates using trial data on the BADS and HADS-D, as well as a range of possible effect size estimates. Estimates do not include expected attrition. Power analyses were conducted based on the primary two-group comparison of interest. An alpha of 0.05 and a beta of 0.80 were used. BADS = Behavioural Activation for Depression Scale; HADS-D = Hospital Anxiety and Depression Scale-Depression

**Raw effect size estimates**			
**Measure**	**AP vs AE**	**AP vs WL**	**AE vs WL**
BADS total	*N* = 138 (69 per group)	*N* = 52 (26 per group)	*N* = 328 (164 per group)
HADS-D	*N* = 970 (485 per group)	*N* = 14 (7 per group)	*N* = 12 (6 per group)
**Range of estimates of effect**			
**Estimate**	**AP vs AE**	**AP vs WL**	**AE vs WL**
*d* = 0.30	*N* = 350 (175 per group)	*N* = 350 (175 per group)	*N* = 350 (175 per group)
*d* = 0.40	*N* = 198 (99 per group)	*N* = 198 (99 per group)	*N* = 198 (99 per group)
*d* = 0.50	*N* = 128 (64 per group)	*N* = 128 (64 per group)	*N* = 128 (64 per group)
*d* = 0.60	*N* = 90 (45 per group)	*N* = 90 (45 per group)	*N* = 90 (45 per group)
*d* = 0.70	*N* = 66 (33 per group)	*N* = 66 (33 per group)	*N* = 66 (33 per group)
*d* = 0.80	*N* = 52 (26 per group)	*N* = 52 (26 per group)	*N* = 52 (26 per group)
*d* = 0.90	*N* = 42 (21 per group)	*N* = 42 (21 per group)	*N* = 42 (21 per group)
*d* = 1.00	*N* = 34 (17 per group)	*N* = 34 (17 per group)	*N* = 34 (17 per group)

#### Harms

Potential harms (worsening HADS scores or changes in reporting of suicidality) of either BA group were explored. Full data are presented in Additional File 2*: *Tables S8 and S9*.* In brief, even when accounting for missing data [59], proportion of those improved in terms of HADS-D scores were greater in both the AP and AE groups. There appeared to be no evidence that participation influenced reporting of suicidal ideation.

### Challenges in trial implementation

As part of the trial, a formal steering committee consisting of clinicians, researchers, and an ABI survivor was formed to oversee recruitment and study progress. An overview of challenges in implementing the trial from the steering committee’s perspective is presented in Additional File 2*: *Document S5*.* In brief, challenges included efficient recruitment through NHS services relative to ABI charities, online intervention delivery resulting in less fatigue due to travel but affecting group social interactions, ABI participants experiencing difficulties with recalling specific group experiences during the qualitative interview at Time 3, and COVID-19 affecting the range of activities participants could partake in throughout the trial.

## Discussion

This study examined the feasibility, acceptability, and, as a secondary aim, the potential efficacy of two 8-week BA groups in ABI. In line with “Traditional” BA, the AP group emphasised planning reinforcing activities outside of the weekly sessions alongside strategies to mitigate planning difficulties post-ABI. The AE group, or “Experiential BA”, emphasised engaging in pleasurable activities within weekly sessions. Both groups were feasible and acceptable, even when delivered online, and had high fidelity. In the AP group, 58.33% of individuals had MCID improvements in activity levels from baseline to post-group, compared to 50% AE and 38.5% WL. Only AP participants showed within-group statistically significant activity level improvements that were maintained at 1-month. On secondary efficacy outcomes, both AP and AE participants had significantly lower depression scores post-intervention versus WL, though only AP participants demonstrated reductions in anxiety, difficulties with motivation, and the inhibiting effects of uncertainty. Secondary improvements in both groups were maintained 1-month post-intervention.

### Interpretation

#### Comparison with previous BA research

This study aligns with one-to-one BA interventions in stroke and neurological populations more broadly [14, 15]. Such benefits have not, however, been reported in all studies (e.g., Gertler and Tate [60], Hart et al [25]). Given most evidence of BA is in stroke, a study strength is its inclusion of a wider variety of individuals with ABI, though further work with larger samples is required to examine whether aetiology is a factor in feasibility, acceptability, and outcomes.

#### Primary objective—feasibility and acceptability outcomes

Our study met the target sample size and had low attrition rates (< 20% across all arms). The WL design that allowed all participants access to an intervention is potentially relevant to these good retention rates and is certainly consistent with many health services that have waiting periods between assessment and intervention. In taking forward feasibility results to an effectiveness trial, the re-randomisation element also allows for a more efficient design, where all participants recruited can fulfil the important roles of a comparison group (WL) and as a member of either AP or AE groups and thus increase group size. To further enhance recruitment, recruitment via self-referrals and ABI charity routes is potentially more efficient for future trials, as found in previous BA studies in stroke [14].

Group size was important for acceptability; while smaller groups facilitated greater companionship, groups with too few group members were sometimes perceived as less beneficial and therefore a larger minimum group size may be warranted in future trials. Care should be taken when forming randomised groups with participants at various timepoints post-ABI as this may affect group cohesion. Participants noted that including content on how ABI varies by diagnosis and time post-event within session may be beneficial in ensuring group cohesion.

Both groups received high ratings of helpfulness and enjoyability. Although we incorporated strategies to mitigate the cognitive demands of planning activities in the AP group, some participants reported difficulties in concentrating throughout sessions and remembering details about session content. There was however no overall statistical difference in self-reported “cognitive barriers” between groups, suggesting that the demands of the AP group were not generally greater than the types of activity/social groups often run by e.g., charities at the recruited sample size. Exploring whether cognitive demands differ in group BA, compared to one-to-one BA, is warranted.

Though qualitative data indicated that online groups were preferred over in-person groups due to no travel time, a greater number of attention, fatigue, and organisational—as well as technological—barriers were reported for online groups. The degree to which these barriers reduce in a few years’ time (as experience of videoconferencing technology increases) is an interesting question. Whilst in-person groups may remain essential for some individuals to benefit, there is no doubt that online delivery has great potential to increase accessibility and save costs to both therapists and patients in terms of travel.

### Secondary objectives—exploration of effects

To our knowledge, this is the first study to investigate *group* BA in ABI and the first to compare “Traditional” BA (AP) to an alternative method of delivery (“Experiential” BA; AE) and WL controls. Given our primary aims were feasibility and acceptability, our study was not necessarily powered to detect differences in outcome measures and therefore exploratory analyses here should be interpreted cautiously. With our recruited sample size, only AP participants showed significant within-group BADS improvement. In light of our power analyses for future trials, our sample was insufficient to detect a statistically significant Time × Group interaction. Therefore, replication of mean changes in activity levels in a powered sample is warranted.

In contrast, with the recruited sample size, the AP group was statistically superior to AE and WL in terms of reductions in depression symptoms (HADS-D), anxiety (HADS-A) and intolerance of uncertainty (IU-SF Inhibitory Anxiety), and improvements in motivation (BMQ-S), with over 50% of AP participants demonstrating MCID improvements in anxiety and depression. All of these gains were well maintained one month following therapy cessation, indicating that these may be stronger effects observable in small sample sizes. Given that the current study was not powered to examine these secondary outcome measures, replication of these secondary effects in a newly recruited sample is required. Longer follow-up periods may also be beneficial to assess longer-term maintenance of gains.

There are many potential reasons why, within a BA framework, the desired intervention *outcome* (improvements in mood) was reliably observed whilst the proposed *mechanism* (increased activity engagement) was not statistically distinguishable from WL. One possibility is that the relevant measures have different sensitivities; greater activity level increases were enough to reduce depression, anxiety, and related outcomes but were themselves insufficient to cross a statistical threshold with the recruited sample. It is also possible that activity levels increased (at the very least *attending* the groups and activities within them probably represented an increase for many participants) but that participants were less sensitive to these changes in self-reports relative to mood. Another possibility is that group attendance itself improved mood independently of any activity level change. In an early characterisation of BA, exactly this priming of the mood-activity cycle was posited—“energy level” increases were a prerequisite for activity level increases [61]. If this is the case, changes in activity levels may lag the mood changes but may remain important for the maintenance of mood gains. The results are certainly useful in selecting the most important efficacy outcome measures for subsequent trials and the time-course for assessing change. In terms of enhancing likely effect of groups like AP, greater incorporation of core values assessment throughout may result in greater long-term sustained increases in activities [34, 62].

AE-style groups that have no explicit therapeutic agenda are far less investigated than conventional therapeutic groups in terms of potential mood improvements. These groups can provide a safe and predictable milieu where individuals can form a continuous support network [26, 29] and meet others experiencing similar difficulties to help reduce stigma of ABI [63] that might contribute to activity level increases. Though there was no increase in BADS scores at the recruited sample size, depression symptom reductions were observed and maintained at 1-month following group end, suggesting some lasting impact. Again this is consistent with the notion of an “energy level” boost potentiating subsequent change. Whilst the anxiety and motivation results here certainly suggest that the AP group produced greater benefits, the possibility that AE-like groups could be a useful “BA” intervention for those with marked cognitive problems merits further exploration in a powered sample.

There may be a number of reasons for the apparent superiority of the AP group in mediating greater mood improvement. Firstly, the AP group explicitly encourages participants to plan and engage in between-session positive activities. Second, it incorporates goal management techniques to mitigate barriers to activity engagement. Thirdly, the achievement of these between-session activities may increase a sense of mastery/control that in turn contributed to mood gains [64, 65]. Finally, it is also possible that the AP group serves to modify dysfunctional beliefs and attitudes that are linked with low mood. The rationale for BA over cognitive approaches was never that unhelpful beliefs did *not* affect mood, but rather these beliefs could be equally or *better* modified via behavioural experimentation than explicit cognitive restructuring [66–68]. Changes in motivation, anxiety, and coping with uncertainty in AP participants may reflect changes in unhelpful beliefs about activities. There are likely several factors that explain greater improvements in AP versus AE participants that should be explored in a sufficiently powered sample.

### Potential sources of bias

As with all psychological intervention research, interventions can only be single-blinded and therefore completely unbiased effects are not possible. As both interventions were delivered by one person, this minimised the issue of whether “therapy differences” are attributable to “therapist differences” (e.g., confidence, experience). However, this raises the question of whether facilitator beliefs about likely efficacy of each group played a role. Both groups were designed to maximise effectiveness, discussed with a clinical supervisor, and delivered in the knowledge that fidelity would be assessed—which was, as discussed, rated as very high. An important issue for subsequent trials would be maximising facilitator and participant “buy-in” to both approaches to not skew favour toward either approach. There is always the risk that participants who consent to take part in research are not fully representative of the wider population who may be referred to groups in clinical services. In addition, those who self-referred by responding to online invitations may differ in a number of ways from others living with ABI.

### Generalisability

Generalisability has benefitted from using a mixed-methods design, in accordance with MRC recommendations [69]. The combination of methods provides richer acceptability and feasibility data and highlights areas to augment in future trials.

A study strength is that circumstances necessitated a variety of recruitment routes, from clinician referrals in NHS services, through charities and from social media self-referrals that can be useful to use in future trials. The blocked randomisation ensured that potential participant differences due to recruitment route would not have a disproportionate impact on any group. Whilst underpowered to examine quantitative outcome differences, the qualitative data at least suggest that both interventions were well-received.

Participants were mainly in the chronic stages of ABI. Earlier stages are associated with greater frequency of impairments and impact on function, which can limit intervention engagement [70]. Some may be less “ready” to accept emotionally focused interventions in early ABI [14]. However, a previous BA intervention in early (within 4 months) stroke reported benefits [71]. ABIs are increasingly being recognised as a chronic condition that require long-term emotional support [72, 73]. It is a study strength to demonstrate that emotional improvement can occur in chronic ABI and greater recruitment from this population may be warranted in community-based NHS settings.

It is likely that improvements in group outcomes in part rely on the degree of cohesion among BA group members. Our trial conducted randomisation at the individual level and therefore pre-selection of similar participants to either group (e.g., in terms of demographics and ABI severity/type of impairments) was not possible. However, our qualitative data indicated that some participants preferred groups comprised of individuals with similar experiences/needs. Future trials should therefore evaluate whether alternative randomisation methods (e.g., cluster randomisation) may be appropriate and incorporate measures of group cohesion as an outcome mediator. Of course, in clinical settings, there is often a balance between using waiting lists to accumulate group participants with similar needs and imposing unduly long waiting times. For this reason, individual therapy provision, or the use of rolling admissions into formerly “static” BA groups to ensure earlier access to an intervention, may be more practical in some cases. Groups such as the Experiential BA group may lend itself to rolling recruitment better than a Traditional BA group that could perhaps be used as a method of positive benefit until a more static Traditional BA group begins.

In protocols for future trials, it may be worth explicitly incorporating how to manage participant delays between baseline assessment and intervention start that strikes a balance between internal and external validity. In clinical practice, participant delays in starting groups (e.g., due to illness, personal circumstances, or return to work dates) may occur occasionally and sometimes intentionally (i.e., in pre-selecting participants who are likely to have good group cohesion). Given we observed three such delays due to personal circumstances within our study, it is likely that a definitive trial will encounter similar situations. One solution could be to pre-specify fixed time windows between baseline assessments and intervention start.

With a mild to moderately depressed sample, it is unknown whether results extend to those with severe depression and therefore further evaluation in severely depressed ABI samples may be warranted. Some participants stated that discussing highly personal and emotionally challenging content was not well-suited to groups. One-to-one support may be preferred in those with more severe depression [74].

Based on data from participants with aphasia, dedicated groups for those with non-fluent aphasia are likely to have greater acceptability [75, 76]. In contrast, people with fluent aphasia given appropriate support and availability of suitable materials appear to benefit from both approaches in our study, though further piloting prior to a definitive trial is needed.

Generalisability must be considered in light of in-person vs. remote delivery. For the former, transport was a considerable barrier for many participants. Transport and access-related barriers relate to community integration and well-being in ABI [77] and are in part responsible for activity level reductions [78, 79]. In-person study results may only be generalizable to those capable of travelling independently or who have carer support in accessing transport; hence, a definitive trial should collect these details alongside recruitment rates.

For online sessions, trading-off limitations (e.g., creating a cohesive group atmosphere online) against higher attendance was preferred by many. Participants with greater physical disabilities and those who found travel too cognitively demanding/tiring particularly benefited. Some online participants, due to service provision differing across regions, could *only* access groups via videoconferencing. It is certainly possible that our online recruitment benefitted from COVID-19 pandemic lockdowns; it was “something to do” when many other activities and in-person socialisation were restricted. It will be interesting to compare online recruitment rates at times when such restrictions are absent. It seems likely, however, that online and hybrid interventions will become more common given growing evidence of efficacy [80–83].

### Limitations

Study feasibility and acceptability are relatively robust *in the current sample.* Though online recruitment enhanced sample diversity relative to in-person recruitment, this still produced a greater number of White British participants relative to UK population data. Hence, generalisation of results to samples more representative of the UK population should not be assumed. Effect size estimates have wide confidence intervals, making it difficult to interpret a true effect of either intervention. Follow-up data at only 1-month post-intervention limits investigation of longer-term effects. The modest sample size prevented meaningful subgroup comparisons between participants with differing ABI severity levels (e.g., mild vs moderate-to-severe TBI) that may relate to degree of acceptability and benefit from the trial. Finally, not recruiting from NHS sources throughout the entire trial limits implementation conclusions for groups in NHS settings with individuals closer to their ABI date and potentially experiencing greater symptom severity, though a detailed account of implementing individual BA in stroke is available [14].

### Summary of recommendations

Based on quantitative and qualitative data, as well as steering committee perspectives, recommendations for changes in trial design, recruitment methods, data collection, intervention design, and study outcomes may be beneficial to consider in a main trial (Table 11). Prior to conducting a definitive trial, we recommend evaluating:

1. Literature review on suitable measures of group cohesion in ABI and potential removal of outcomes measures showing little to no response in BA;
2. Evaluating suitability of alternative randomisation methods for group BA to enhance group cohesion;
3. Replication of MCID efficacy estimates in an independently recruited sample, especially through UK NHS ABI services;
4. Further feasibility testing of the BA groups in ABI survivors with aphasia;
5. Conducting PPI consultations on refining AP group session materials so as to reduce session content;
6. Conduct economic analysis of costs associated with groups conducted in NHS and community settings.

**Table 11:** Recommendations for review prior to implementation and conducting an effectiveness trial based on pilot feasibility data

Protocol aspect	Recommendations	Type of review recommended
Trial design	• Retain Waitlist Control design • Include additional participant support check-ins for Waitlisted participants • Retain online delivery	Staffing costs review, Steering Committee review
Recruitment	• Recruit between acute and community services and chronic samples • Enhance social media recruitment and self-referrals • Clearer advertisement of potential benefits of both BA groups • Identify methods to enhance sample representativeness	PPI consultation, clinician consultation
Data collection	• Include further measures to characterise cognitive ABI symptoms in baseline assessment • Include 3 month or longer follow up • Remove exit interview and include additional questions in PSQ • Qualitative data collected at all follow-up time points	Staffing costs review, literature review
Interventions	• Retain 8 sessions • Review suitability of AP group content • Adapt training manual for facilitator on ways to create therapeutic milieu online • Consider additional co-facilitator	PPI consultation, staffing costs review, Steering Committee consultation
Outcomes	• Include measures of group cohesion • Include measures of positive and negative self-beliefs • Consider self-report executive function measures • Review choice of primary outcome measure (activity levels versus mood)	Literature review, Steering Committee consultation

## Conclusions

In summary, we demonstrate that two methods of delivering Behavioural Activation are feasible and acceptable, with both showing promise in improving activity levels and mood relative to Waitlist Controls in a high-quality randomised controlled design. Benefits of the interventions outweighed reported harms. “Traditional” BA seems to affect motivational processes and enhance confidence relative to “Experiential” BA. Further delineating how group processes contribute to activity levels and mood, and methods of facilitating group cohesion in future intervention studies, is warranted .

### Supplementary Information

**Additional file 1.** Contains a CONSORT checklist with corresponding page numbers for the MAPLES trial.

**Additional file 2.** Contains materials used throughout the study. (**Document S1.** Fidelity Checklists. **Document S2.** Post-Study Questionnaire. **Document S3.** Exit Interview), additional supporting data (**Table S1.** Reasons for Trial Exclusion. **Table S2.** Participants Randomised per Recruitment Source. **Figure S1.** Intervention Attendance by Study Wave. **Figure S2.** Reasons for session non-attendance. **Table S3.** Summary of Credibility Perceptions of Intervention Groups. **Table S4.** Example quotes for Acceptability Themes. **Document S4**. Supporting Participants with Aphasia. **Table S5.** Summary of Recommendations for Supporting Aphasia. **Table S6.** Full results from mixed-effects models. **Figure S3.** Visualisation of Mean Differences for Study Outcomes. **Table S7.** Clinically Meaningful Improvements on Study Measures. **Table S8.** Complete Case Summary of Worsening on Mood Measures. **Table S9.** Sensitivity Analysis on Worsening on Mood Measures. **Document S5.** Challenges in Trial Implementation).

## Data Availability

The dataset supporting the conclusions of this article is available in the study-specific Open Science Framework repository: http://osf.io/e5btr The corresponding author (AK1) may be contacted with any data requests.

## Abbreviations

ABI: Acquired Brain Injury
AE: Activity Engagement group
AP: Activity Planning group
BA: Behavioural Activation
BADS: Behavioural Activation for Depression Scale
BIS/BAS: Behavioural Inhibition/Behavioural Activation Scale
BMQ-S: Brain Injury Rehabilitation Trust Motivation Questionnaire-Self
CBT: Cognitive Behavioural Therapy
CEQ: Credibility and Expectancy Questionnaire
HADS: Hospital Anxiety and Depression Scale
ICC: Intraclass Correlation Coefficient
IES-R: Impact of Events Scale-Revised
IU-SF: Intolerance of Uncertainty Scale-Short Form
MCID: Minimal Clinically Important Difference
MOM-POPS: Modified Outcome Measure-Participation Objective, Participation Subjective
MOT-Q: Motivation for Traumatic Brain Injury Rehabilitation Questionnaire
PMM: Predictive Mean Matching
PSQ: Post Study Questionnaire
SCS: Sense of Control Scale
SEM: Standard Error of Measurement
SHAPS: Snaith-Hamilton Pleasure Scale
TBI: Traumatic Brain Injury
VESPAR: Verbal and Spatial Reasoning Task
WL: Waitlist control group

## Footnotes

1 This included traumatic brain injury, stroke, anoxia/hypoxia, encephalitis and other brain infections, and resected brain tumours.
