# Supplementary material for: Mood, Activity Participation, and Leisure Engagement Satisfaction (MAPLES): results from a randomised controlled pilot feasibility trial for low mood in acquired brain injury
Source: BMC Med. 2023 Nov 16;21:445. doi: 10.1186/s12916-023-03128-7 (PMC10655452; doi:10.1186/s12916-023-03128-7)
Supplement: Supplementary file 2 — Additional file 2. Contains materials used throughout the study. (Document S1. Fidelity Checklists. Document S2. Post-Study Questionnaire. Document S3. Exit Interview), additional supporting data (Table S1. Reasons for Trial Exclusion. Table S2. Participants Randomised per Recruitment Source. Figure S1. Intervention Attendance by Study Wave. Figure S2. Reasons for session non-attendance. Table S3. Summary of Credibility Perceptions of Intervention Groups. Table S4. Example quotes for Acceptability Themes. Document S4. Supporting Participants with Aphasia. Table S5. Summary of Recommendations for Supporting Aphasia. Table S6. Full results from mixed-effects models. Figure S3. Visualisation of Mean Differences for Study Outcomes. Table S7. Clinically Meaningful Improvements on Study Measures. Table S8. Complete Case Summary of Worsening on Mood Measures. Table S9. Sensitivity Analysis on Worsening on Mood Measures. Document S5. Challenges in Trial Implementation). [file 12916_2023_3128_MOESM2_ESM.docx]

***Additional File 2***

*Document S1: Fidelity Checklists*

**MAPLES Pilot Feasibility Trial Fidelity Assessment Checklist**

**Activity Planning Group**

Rating Instructions: Upon listening to each audiorecording for each session, rate each aspect on whether they occurred as below:

0: Component not present/not attempted

1: Facilitator attempted component partially/questionably

2: Facilitator covered component and relevant content

A new checklist should be completed for each cohort of the Activity Planning Group.

**Session 1: Introduction to Group Therapy
Cohort Number:**

**Date:**

____ Introduction to the group structure

____ Introduction of group members

____ Introduce link between activity level and mood

____ Discussed examples as relevant to participants

____ Rationale of behavioural activation

____ Education on executive function difficulties in brain injury

____ Introduce concept of absentmindedness

____ Introduce concept of mood and mind monitoring

____ Explained and set homework tasks

**Session 2: Identifying Enjoyable Activities**

**Cohort Number:**

**Date:**

____ Reviewed mood and mind monitoring homework from last week

____ Discuss relationship between mood and attention for each group member

____ Introduce value-based activity planning

____ Discussed examples as relevant to participants

____ Introduce Activity Wheel to identify core values

____ Introduce link between core values and personal goals

____ Participants identify short-term goals within the group and long-term goals

____ Participants begin activity scheduling and learn to break down activity into steps

____ Explained and set homework tasks

**Session 3: Changing Habits and Planning Pleasurable Activities**

**Cohort Number:**

**Date:**

____ Reviewed activity scheduling homework from last week

____ Provide example of how absentminded errors occur in daily life

____ Introduce concept of the automatic pilot

____ Discussed examples as relevant to participants

____ Introduce identifying personal triggers to absentminded errors

____ Participants identify personal triggers to absentminded errors within group

____ Participants schedule another activity and break down activity into steps

____ Explained and set homework tasks

**Session 4: Goal Review and Balancing Enjoyable and Routine Activities**

**Cohort Number:**

**Date:**

____ Reviewed activity scheduling homework from last week

____ Review homework on identifying personal triggers for absentminded errors

____ Education on preventing personal triggers

____ Discussed examples as relevant to participants

____ Participants create personalised strategies to prevent triggers

____ Participants review progress on goals from session 2

____ Group discussion on whether activities in day-to-day life align with values/goals

____ Introduce concept of balancing between routine and enjoyable activities

____ Participants schedule another activity and break down activity into steps

____ Explained and set homework tasks

**Session 5: Identifying Solutions to Goal Attainment**

**Cohort Number:**

**Date:**

____ Reviewed activity scheduling homework from last week

____ Review homework on preventing personal triggers

____ Education on falling into avoidance patterns

____ Discussed examples as relevant to participants

____ Participants create personalised strategies to reverse avoidance patterns

____ Participants review concept of the automatic pilot

____ Participants learn STOP acronym to prevent distraction from goal attainment

____ Participants practice using STOP acronym

____ Participants schedule another activity and break down activity into steps

____ Explained and set homework tasks

**Session 6: Increasing Mastery and Managing Fatigue**

**Cohort Number:**

**Date:**

____ Reviewed activity scheduling homework from last week

____ Review homework on reversing avoidance patterns

____ Education on importance of improving mastery and risks of plateauing

____ Discussed examples as relevant to participants

____ Participants identify “warning signs” of plateauing

____ Education on different types of fatigue post-brain injury

____ Participants identify personalised triggers to fatigue

____ Participants develop personalised strategies to manage fatigue

____ Participants schedule another activity and break down activity into steps

____ Explained and set homework tasks

**Session 7: Active Approaches to Engagement**

**Cohort Number:**

**Date:**

____ Reviewed activity scheduling homework from last week

____ Review homework on attempting strategies to prevent fatigue

____ Education on importance of social relationships in maintaining a good mood

____ Discuss examples as relevant to participants

____ Education on active versus passive approaches to activity scheduling

____ Discussion on personal barriers to taking an active approach

____ Participants practice in session how to initiate a social activity

____ Participants list benefits of the active approach and drawbacks of passive approach

____ Participants schedule another activity and break down activity into steps

____ Explained and set homework tasks

**Session 8: Relapse Prevention**

**Cohort Number:**

**Date:**

____ Reviewed activity scheduling homework from last week

____ Review homework on active and passive approaches

____ Participants identify “take home” message from group

____ Review behavioural activation model from Session 1

____ Review value-based activity planning from Session 2

____ Review concept of automatic pilot from Session 3

____ Review concept of personalised triggers from Session 4

____ Review STOP acronym from Session 5

____ Review importance of increasing mastery from Session 6

____ Review importance of taking an active approach to activities from Session 7

____ Review importance of breaking down activity into manageable steps

____ Education on importance of preventing relapse into lower activity levels

____ Participants identify personal triggers that may cause a relapse

____ Participants create personalised strategies to prevent relapse triggers

____ Participants schedule another activity and break down activity into steps

**MAPLES Pilot Feasibility Trial Fidelity Assessment Checklist**

**Activity Engagement Group**

Rating Instructions: Upon listening to each audiorecroding for each session, rate each aspect on whether they occurred as below.

The Activity Engagement Group is flexible by nature, and this should include:

- No mention behavioural activation/activity scheduling
- No encouragement of direct planning beyond the group
- Facilitator encouragement of engagement on activities within the group
- Facilitator encouragement of participant autonomy of activities chosen
- Non-linear structure of groups

For the first two components, rate as below:

2: Component was not present

1: Facilitator attempted component partially/questionably

0: Facilitator covered component adequately

For the last three components, rate as below:

0: Component was not present

1: Facilitator attempted component partially/questionably

2: Facilitator covered component adequately

A new checklist should be completed for each session of each cohort of the Activity Engagement Group.

**Cohort Number:**

**Session Number:**

**Date:**

____ No mention of behavioural activation/activity scheduling

____ No encouragement of direct planning beyond the group

____ Facilitator encouragement of engagement on activities within the group

____ Facilitator encouragement of participant autonomy of activities chosen

____ Non-linear structure

Due to the broad criteria, examples of the above are below:

*No mention of behavioural activation/activity scheduling*

2: Component not mentioned at all

1: Facilitator occasionally mentions behavioural activation and the importance of scheduling in activities throughout the week

0: Facilitator explains what behavioural activation is in depth and how activity scheduling relates to mood

*No encouragement of direct planning beyond the group*

2: Component not mentioned at all

1: Facilitator encourages a participant to plan something beyond the group in session

0: Facilitator explains the importance of learning to plan in activities as a concept and encourages planning more than once in session

*Facilitator encouragement of engagement of activities within the group*

2: Facilitator encourages participant in completing the activity/encourages socialising in session, and/or provides support for completing an activity, and/or helps explain how to set up an activity to participants

1: Facilitator briefly explains an activity or does not explain an activity adequately/little encouragement of socialisation

0: No encouragement present

*Facilitator encouragement of participant autonomy within the group*

2: Facilitator asks/encourages participant to choose what they want to do/responds appropriately when a participant requests to do a different activity

1: Facilitator responds to participants asking to do something differently within session, but does not follow through with switching

0: No choice/facilitator refutes someone wanting to choose to complete a different activity

*Non-linear structure*

2: If participants wishes to complete a different activity within session, facilitator switches the activity/moves on to a different activity or discussion; and/or facilitator offers to complete an activity in another session; otherwise if session proceeds as planned rate as 2

1: Facilitator acknowledges request for a new activity in group but does not comply

0: Facilitator does not allow a switch between session activities

*Document S2: Post-Study Questionnaire*

**MAPLES Post-Study Questionnaire**

This questionnaire is just asking you your thoughts on participating in the MAPLES groups. We very much welcome all feedback. Because this is a feasibility study we are trying to figure out what can be done better in the future. So, any feedback, whether positive or negative, is important.

There are no right or wrong answers. I would like you to be as honest and open as you can, although you are always free to not answer questions if you do not want to. Please let me know throughout the interview if it is not clear what I am asking.

To protect the privacy of other group members, I’ll ask that you not mention any of the names of the people in your group.

If at any point you are feeling tired or would like to take a break at any time, please let me know.

Do you have any questions?

Randomisation Group (*if participant is not sure, no need to probe to determine if on WL*)

1. Activity Planning Group (3) Waitlist to Activity Planning Group
2. Activity Engagement group (4) Waitlist to Activity Engagement Group
3. Did you like the idea of a study session focused on activity level? We would like your opinion about the focus on activities, as compared to emotions, brain injury, or any other topic. (Yes or No)

(*If needed: can you give me your general thoughts on participating in the group?*)

1. Do you see any benefits to being part of group sessions? (Yes or No)

Comments:

1. On a scale of 1 to 10, how ***helpful*** did you find your group?

**1 2 3 4 5 6 7 8 9 10**

Comments:

(*Prompts – use to facilitate elaboration on rating scale above)*

- Was there one session that you found really helpful?
- Is there something from the group that you found really motivating?
- Was there one session that you didn’t think would be helpful for you at all?

1. On a scale of 1 to 10, how ***enjoyable*** did you find your group?

**1 2 3 4 5 6 7 8 9 10**

Comments:

1. I have here a list of barriers that may have affected participation in the group. Please tell me which barriers you had with a yes or no. After we have gone through them, then I will ask you why they were barriers.
2. **Motivation 4. Time 7. Effort**
3. **Mood 5. Technology 8. Physical**
4. **Organisation 6. Memory 9. Attention**

**10. Fatigue**

Please add in any comments/follow up to explain the answers above:

Was there anything else not mentioned above that made it difficult for you to attend the group sessions? For example, health-related difficulties, or other people needing the computer?

*(if a participant did not list any barriers, skip this question)*

1. Is there anything you can think of that the research team can do to help you overcome these barriers for future group sessions?
2. Do you think that regular contact with the research team by telephone or email was helpful? (Yes or No)

Comments:

1. Did you find having the group conducted online was useful? Do you see any benefits or challenges? (Yes or No)

Comments:

*(prompts – use to expand on answer to above question if needed)*

- Do you think the groups would be the same in person?
- Do you think that having the groups online was better for your schedule?
- Did it take extra effort to take part in the groups online?

1. Since completing the study, have you noticed any change in the following areas? Please pick the response that you think is the most honest to you. When in doubt, go with your gut response. Please pick a number or category first, then you will have a chance to explain more if you would like to.

|  | **A lot worse (1)** | **A bit worse (2)** | **No change (3)** | **Improved a bit (4)** | **Improved a lot (5)** |
| --- | --- | --- | --- | --- | --- |
| **Your memory** |  |  |  |  |  |
| **Your concentration** |  |  |  |  |  |
| **Your ability to plan/ organise yourself** |  |  |  |  |  |
| **Your spatial awareness** |  |  |  |  |  |
| **Your motivation** |  |  |  |  |  |
| **Your mood** |  |  |  |  |  |
| **Your number of social interactions** |  |  |  |  |  |
| **Or something else** |  |  |  |  |  |

1. Is there something important to you that you feel wasn’t addressed well enough or covered in the group?
2. Do you feel that there are any other improvements not already mentioned that could be made to the group? Please be as detailed as possible.

*Document S3: MAPLES Study Exit Interview*

**MAPLES Study Exit Interview**

*At the start of the audiorecording, please state your name and today’s date, along with who you are interviewing by participant number.*

Participant Number:_______

This Exit Interview covers questions about specific aspects of your group. You will have an opportunity to expand your thoughts in greater detail. Just like in the Post-Study Questionnaire, it is really important to get all sorts of feedback, both positive and negative.

I’ll remind you again not to mention any of the names of the people in your group for confidentiality.

Do you have any questions?

Ok, let’s begin.

**Feedback on Homework in Activity Planning Group**

**(Ask the below only if participant took part in the Activity Planning Group)**

Did you feel that the homework was relevant, manageable, and helpful? Why or why not?

*(Homework tasks – use to ask specifically about each task. Interviewer can share screen for specific content if needed as a guidance)*

- Planning in meaningful/enjoyable activities/break down activities step by step
- Mood and Mind monitoring – reflecting on what you do on a day-to-day basis and how this makes us feel (mood) and how focused we feel (mind)
- Using the ABCs (Antecedent, Behaviour, Consequence – understanding what triggers negative thoughts or feelings or events)
- TRAP/TRACs (Trigger, Response, Avoidance Pattern/Active Coping)
- Using the STOP acronym (Stop, Think, Organise, Proceed)
- Active vs Passive Approaches to Activities

Was there anything that made it difficult for you to complete the homework?

Now, I will ask about the content within each session, rather than the homework.

Did you find that the content within the group sessions was useful to you?

*(if needed, refer to Activity Planning Group discussion guide)*

Were there any sessions that were not very useful to you?

*(if needed, refer to Activity Planning Group discussion guide)*

(*If necessary)*

Can you expand on your answer some more?

**All participants are asked the below**

**Practical Aspects of the MAPLES study**

I am going to ask about some practical aspects of being part of the MAPLES study.

Was there someone in your personal life that affected your participation in the study, either for better or for worse? For example, a partner, closer friend, or family member?

Was there something that happened in the group sessions that made you feel like it wasn’t helpful? For example, your relationship with other group members? Again, please be careful not to mention anyone’s name for confidentiality.

**Facilitator Influence**

Thank you. Now, we are interested in understanding if the facilitator affects how you respond to the group. Remember that any feedback is welcome, both positive and negative.

Do you think the groups would be the same with someone else running the sessions?

What qualities do you think are necessary for a person running this kind of group? If it’s helpful, refer back to other groups you have attended.

Do you think there was something Andrea could have done differently in how she ran the sessions?

*(if they have mentioned it PSQ, refer back to answer here)*

Okay, now I’d like to ask if you felt 8 sessions was enough, or if it was too much, or not enough.

*(prompt)*

- How many sessions would be ideal for you?
- What length of session is ideal for you? For example, 1 hour, 2 hours, etc

Did the current pandemic affect your experience in the group? How?

Do you have any other comments or feedback?

*Reasons for Trial Exclusion*

| **Reason Excluded (*N* = 74)** | **In Person** | **Online** | **Total** |
| --- | --- | --- | --- |
| Did not Meet Eligibility Criteria  No Low Mood/No Low Activity Level  Incapable of Attending to Study Materials  Current Psychotherapy  Suicidality/Risk of Immediate Harm to Self  Does not speak English  Recent Change in Psychotropic Medication | 11  5  4  2  1  1 | 12  1 | 23  6  4  2  1  1 |
| Declined Participation/No Longer Interested | 12 | 10 | 22 |
| No Response after Expression of Interest | 7 | 3 | 10 |
| Other  Mobility Issues Affecting Participation  Client Wants Limited Input in Services  Due to Move away from Study Location  Lives Outside of UK | 1  1  1 | 1 | 1  1  1  1 |

**Table S1**. Summary of reasons for exclusion from the trial. Of note, current psychotherapy was no longer an exclusion criteria following a protocol amendment approved December 2019.

*Number of Participants Assessed vs Randomised per Recruitment Source*

| Assessed (Randomised) | **NHS ABI Services** | **Research Panel** | **Self-referral via Social Media** | **ABI Charity Groups** |
| --- | --- | --- | --- | --- |
| In Person | 58 (19) | 28 (3) | 0 | 3 (3) |
| Online | 0 | 5 (3) | 21 (14) | 30 (18) |
| **Total** | 58 (19) | 31 (6) | 21 (14) | 33 (21) |

**Table S2.** Participants assessed (*N* = 143) versus those randomised (*N* = 60) per recruitment source.

*Intervention Session Attendance by Study Wave*

An overview of session attendance per wave is shown in Figure S1. The pattern of session attendance across study waves was variable, most notably for Wave 3, which was halfway completed when the first UK COVID-19 lockdown was announced (March 23 2020). In the AP group, this resulted in no attendees for Week 5 (the first week following the onset of lockdown) and a participant withdrawing due to security concerns about videoconferencing. In the AE Group, this resulted in only one person in attendance for Weeks 5 and 6 and another group member, who did not wish to attend the group online for Week 5, was hospitalised due to COVID-19 but opted to complete the Time 3 assessment.

**
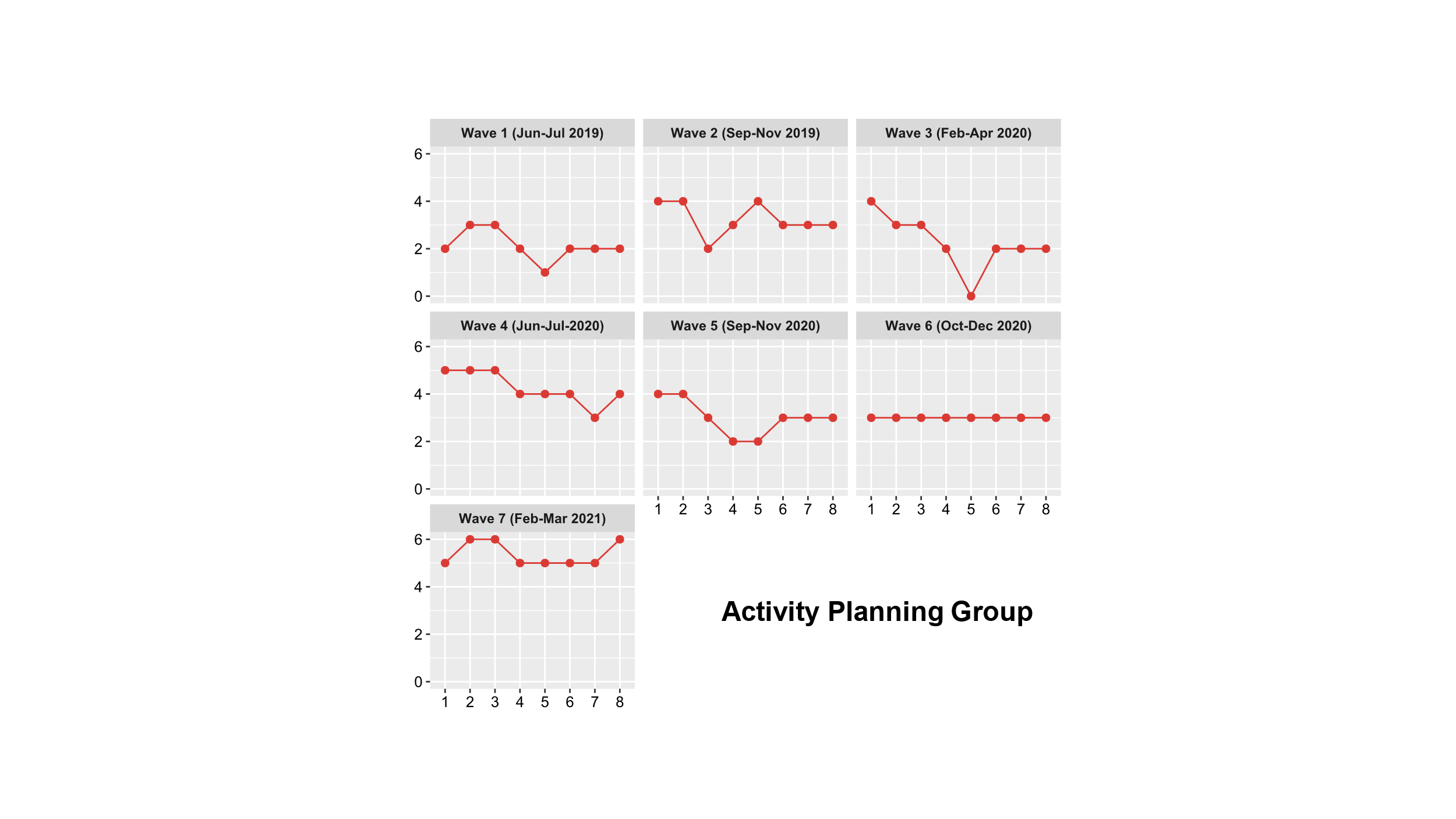

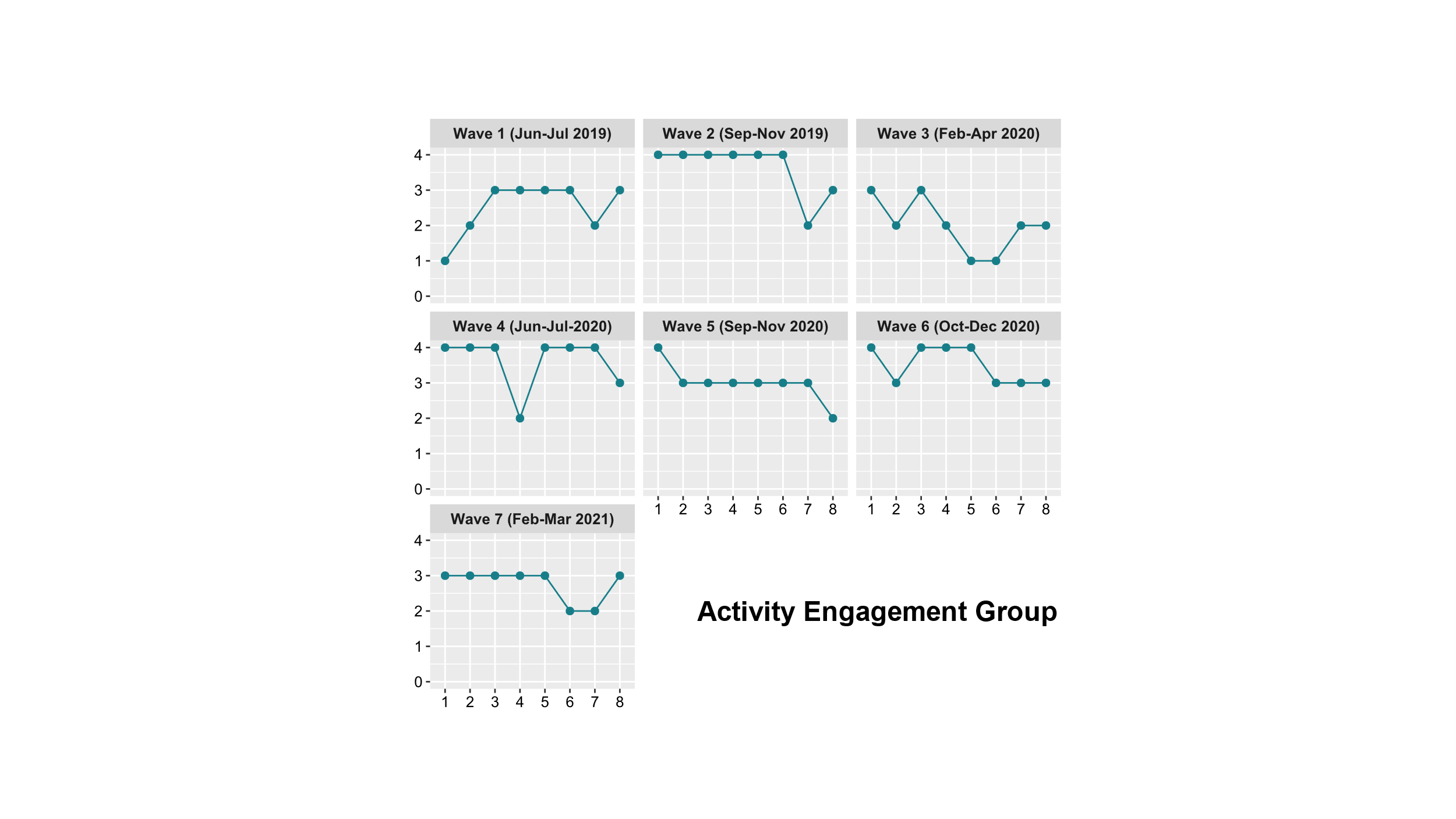
**

**Figure S1.** Total group session attendance per recruitment wave in the AP Group (above) and the AE Group (below). X-axes denote session number, and y-axes number of participants. Waves 1, 2, and the first half of Wave 3 were conducted in person. Waves 4 to 7 were conducted remotely via videoconferencing.

The most commonly missed session was Session 5 in the AP Group (*n =* 7), and Session 7 in the AE Group (*n* = 5). A full list of reasons for non-attendance is shown in Supplementary Figure 2. Generally, the majority of reasons were independent of study factors or group content.


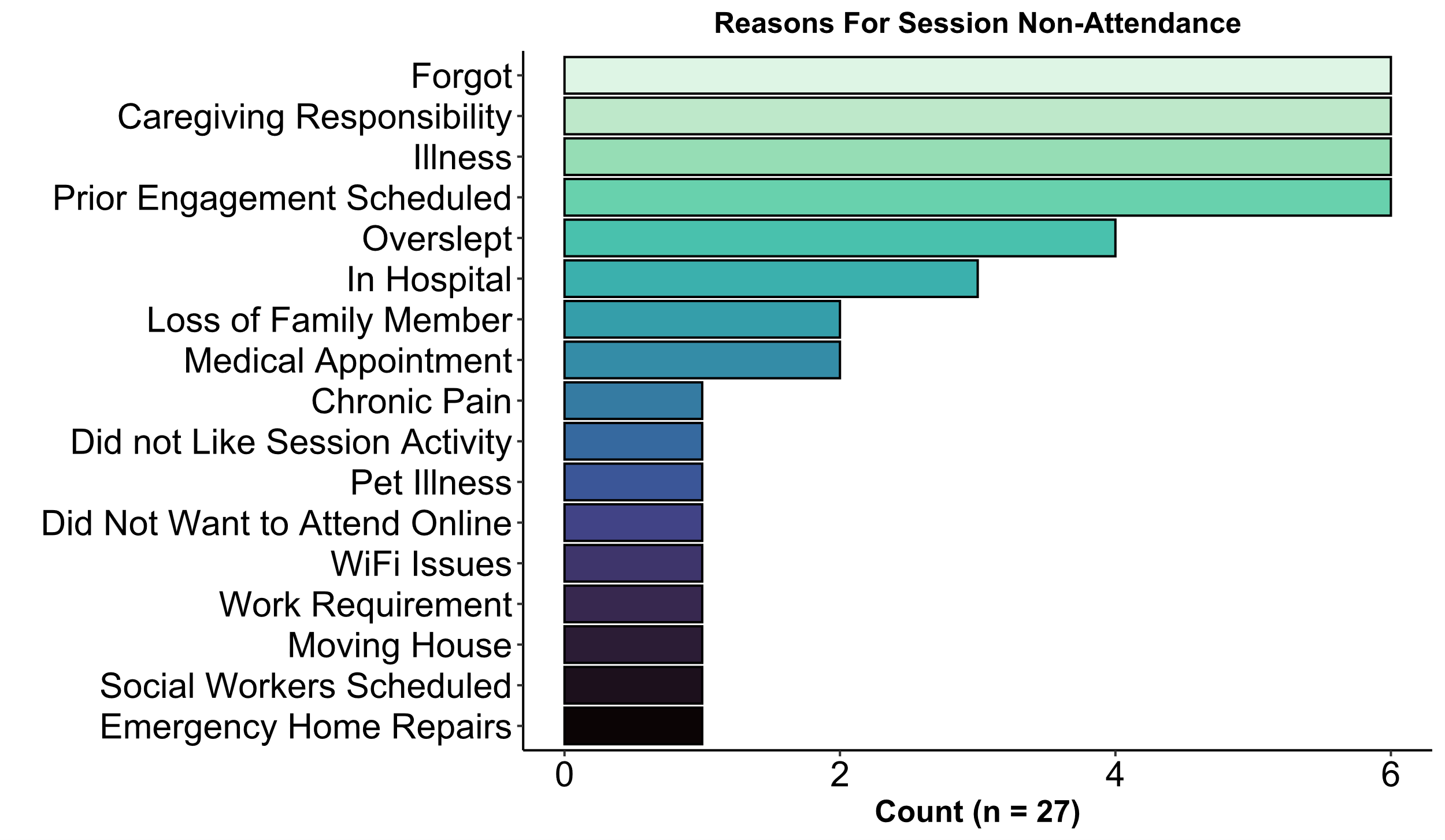


**Figure S2.** Reasons for session non-attendance in the AP and AE Groups. Number on the x-axis indicate the total number of times a reason for non-attendance occurred. A total of 44 sessions were missed across 27 participants.

*Credibility and Expectations of Interventions*

| **CEQ Item**  ***M* (*SD*)** | **Activity Planning Group**  (*n* = 29) | | | **Activity Engagement Group**  (*n* = 28) | | |
| --- | --- | --- | --- | --- | --- | --- |
|  | In Person | Online | Total | In Person | Online | Total |
| How logical does the group offered to you seem? (max 10) | 7.50 (1.51)  Median=7.5 | 7.11 (1.70)  Median=8 | 7.24 (1.62)  Median=8 | 6.45 (2.46)  Median=7 | 6.71 (2.31)  Median=8 | 6.61 (2.33)  Median=7.5 |
| How successful do you think this group will be in improving your activity levels? (max 10) | 7.60 (1.35)  Median=8 | 6.68 (1.86)  Median=7 | 7.00 (1.73)  Median=7 | 6.64 (2.42)  Median=7 | 6.12 (2.52)  Median=7 | 6.32 (2.45)  Median=7 |
| How confident would you be in recommending this group to a friend? (max 10) | 6.90 (1.45)  Median=7 | 6.84 (1.95)  Median=7 | 6.86 (1.77)  Median=7 | 7.00 (1.73)  Median=7 | 6.47 (2.76)  Median=7 | 6.68 (2.39)  Median=7 |
| What percent improvement do you *think* will occur in your activity levels? (max 100) | 60.0 (2.1)  Median=60 | 52.1 (28.8)  Median=50 | 54.8 (26.5)  Median=60 | 52.7 (22.84  Median=50 | 55.9 (26.5)  Median=50 | 54.6 (24.7)  Median=50 |
| How much do you *feel* the group will help you improve activity levels? (max 10) | 6.80 (2.10)  Median=7 | 6.26 (2.18)  Median=7 | 6.45 (2.13)  Median=7 | 6.36 (2.25)  Median=6 | 5.59 (2.45)  Median=7 | 5.89 (2.36)  Median=7 |
| What percent improvement do *feel* will occur in your activity levels? (max 100) | 61.0 (27.7)  Median=75 | 54.3 (28.4)  Median=60 | 56.6 (27.8)  Median=60 | 54.2 (28.4)  Median=70 | 57.1 (25.7)  Median=60 | 56.1 (25.3)  Median=60 |

**Table S3.** Summary of credibility perceptions of the Activity Planning and Activity Engagement Groups, organised by in person and online recruitment as well as combined scores. CEQ Items were given to participants following allocation to either group.

CEQ = Credibility/Expectancy Questionnaire

| **ACCEPTABILITY THEMES** | | | |
| --- | --- | --- | --- |
|  | **Activity Planning Group** | **Example Quotes** | |
| **Group Factors** | **Perceived Benefits and Prerequisites for Success** | | |
|  | - Sharing group materials ahead of each session | *“I was thinking whether there could be um a rough analysis or schedule of what you were going to be done on paper, so that you could then you could read up what it was going to be this time and, you know, look into it in that way.”* Participant Janelle | |
|  | - Providing a summary of session content following session end | “*[The summary gives you] something to reflect back on. And if you’re not sure that reassures you.”* Participant Luka | |
|  | **Suggestions for Improvement** | | |
|  | - Paced Information and Time Management | “*I can't think of anything that you guys could do [better] other than making sure the groups stay on their planned days and times…it's um, then I'm tired, and don't let the group overrun*.” Participant Nelson | |
|  | - Time limits for group discussion relative to session content | “*I think [the facilitator] got a bit stymied by the fact that we were a chatty group. So there were a couple of sessions where we got a bit side-lined...the content ended up being a bit rushed.*” Participant Clara | |
| **Study Factors** | **Perceived Benefits and Prerequisites for Success** | | |
|  | - Wide range of relevant strategies | “*I think that we were focused, you know, is making us do things that we wouldn't normally do…And. And just yeah, developing some tools*.” Participant Oliver  “*It was helpful, but I don't know how helpful in particular for me.”* Participant Jane | |
|  | - Learning from other members’ activities | “*I think it was nice to find out what other people were achieving and how they were progressing throughout the sessions.”* Participant Matthew | |
|  | **Suggestions for Improvement** | | |
|  | - Incorporate more explicit session material on sharing ABI story | “*One thing I would have liked at the beginning of this session or sometime in the sessions, if. We could have been told what the trauma had been to each person participating to know where they were sitting, why they were there and. How it affected them. Bit of past medical history*.” Participant Janelle | |
|  | - Increase session duration/number of sessions, or | *“A few more might have been a bit more beneficial because there are times when one or all of us weren't able to make the meetings and so we missed out a little bit or potentially. And so you I mean, if you if there's only eight and you miss one or two. That's a big chunk of that you might learn lost. Whereas if it's a little bit more then losing a day or two is less of an impact.”* Participant Oliver | |
|  | - Reduce content per session | “I*t might have been easier with the, if the, the video [calls] have been shorter*.” Participant Cory | |
| **Facilitator Factors** | **Perceived Benefits and Prerequisites for Success** | | |
|  | - Sufficient training delivering session materials | *“Obviously there's certain... there's a rulebook of things you have to, that the course of whatever covers. I mean, I should imagine somebody else could do it.”* Participant Henry | |
|  | - Empathic response when activities were not completed | “*I've participated in NHS groups before, and because, um, because they were setting me goals which weren't achievable, they were making me feel bad about myself. I failed because of that, um, But with, uh, the activities that we just did, it didn't matter. If you didn't get your goal, if you took steps and you understood why you didn't reach that goal, that was OK*” Participant Amy | |
|  | **Suggestions for Improvement** | | |
|  | - Communicate session topics using clear language | *“I found some of the…the way it was presented. To me, I’ve just finished an undergrad in psychology so I kind of understand it. I found it was a little bit um confusing because I noticed some of the other members sometimes would go a little bit off tangent, as if they didn’t really understand what exactly was asked of them*” Participant Phoebe | |
|  | - Keep discussions suitably focused | “*I felt like the discussion sometimes wasn’t about what it should’ve been if that makes sense…I think sometimes it could go a little bit off-topic.”* Participant Phoebe | |
|  | **Activity Engagement Group** | **Example Quotes** | |
| **Group Factors** | **Perceived Benefits and Prerequisites for Success** | | |
|  | - Having a variety of activities suitable for different types of ABI | “*When I had the stroke, it paralysed my left side, so doing crafts with my left hand is difficult”* Participant Lila | |
|  | - Group activities were of interest | “*I would say the session I found interesting rather than helpful was the one where we did some games…I think I enjoyed that.”* Participant Angelica | |
|  | **Suggestions for Improvement** | | |
|  | - Prioritise group discussion | “*I would have preferred it to be more of a social group with more talking and discussions and sharing ideas and that sort of thing.”* Participant Ryan | |
|  | - More discussion of ABI | [when asked about sharing information about ABI] “*I think the only thing is, I think it would have helped all of us, not just me, all of us, to have a little understanding of each other from the beginning.”* Participant Eileen | |
| **Study Factors** | **Perceived Benefits and Prerequisites for Success** | | |
|  | - Sharing personal interests with others | “*[The group] allowed me to talk about our interests and learn about others’ interests…it was fun to be part of.”* Participant Eric | |
|  | **Suggestions for Improvement** | | |
|  | - Clear and repeated rationale for potential benefits of group | “*I can’t really remember what the introductory things out of it was, but I didn’t absorb it enough, so I was a bit perplexed about what [the facilitator] and I were hoping to learn from it.”* Participant Lita | |
|  | - Increase session duration | “*I think the only issue is, is when you’re having fun, it goes too quickly.”* Participant Eric | |
|  | - Explicit discussion about mood | “*I think that talking with emotions and being more active with the group, with how they deal with situations or feelings would be beneficial as well*.” Participant Maya | |
| **Facilitator Factors** | **Perceived Benefits and Prerequisites for Success** | | |
|  | - Regularly offer choice in selecting activities | *“[The facilitator] gave us a lot of free rein…but you know, it worked well. If you had a sort of more didactic person who says ‘We’re doing this this week, and that next week’ I wonder if we’d have responded as well.”* Participant Lita | |
|  | - Using low-demand activities that just had a few steps to learn | *“There probably was a few activities where I was a bit like ‘oh um I can’t do this, this isn’t for me’…I think even [the facilitator] was a bit like ‘oh no, this is not good.’ So we had to cut that one off.”* Participant Lawrence | |
|  | **Suggestions for Improvement** | | |
|  | - Group protocol should specify need to adapt sessions based on participant needs | *“It’s trying to deal with a group of people who all have different needs…when I used to attend [NHS brain injury service] they talked about specific things like how the brain worked and what might have happened, and they were talking about people who had different levels.”* Participant Ryan | |
|  | - Fatigue management for in-session activities | *“I found the actual activities extremely tiring…it shouldn’t be very demanding, but, because just how I am, it was.”* Participant Gillian | |
|  | **Both Groups** | | **Example Quotes** |
| **Group Factors** | **Perceived Benefits and Prerequisites for Success** | | |
|  | - Learning from other group members | | “*A lot of the time I saw benefits because of other people, the other members, their experiences I could relate to.”* Participant Luisa |
|  | - Day-before and day-of reminders of sessions | | “*The reminders, um, even the day before reminder is good, but an hour before reminder, that’s, that’s more, even more important to me, because I forget things within an hour or two hours.”* Participant Gillian |
|  | **Suggestions for Improvement** | | |
|  | - Dedicated time for group members to interact without facilitator | | “*I think maybe, not even actually in session, if you get there early and then someone else is there early, you’re not just going to sit there and not speak with them. So you could then, you know, just discuss things with them.”* Participant Jane |
|  | - Adaptable materials for different issues for different ABIs | | “*I think it would be best to have a “pick and mix” example, a different package for those with different difficulties*.” Participant Will |
| **Study Factors** | **Perceived Benefits and Prerequisites for Success** | | |
|  | - Dedicated transport support/ reimbursement for in-person sessions | | “*The [study site] saved us a car parking spot and put our name on it and everything, so there was no problem.”* Participant Henry |
|  | - Regular breaks | | [when asked if the session breaks helped] “*Definitely…the break in the middle was enough to come back feeling refreshed.”* Participant Gillian |
|  | - Remote Delivery | | “*I suppose for me [the group] wouldn’t be more useful in person because then you haven’t got all that ‘oh, I’ve got to then drive here and get to some place that I don’t know.” So that bit was good from my point of view. Doing it online means you don’t have to worry about travelling somewhere and trying to find somewhere to go.”* Participant Lawrence |
|  | **Suggestions for Improvement** | | |
|  | - Increase minimum group size | | “*Our group was supposed to be larger and then we ended up with two in last few [sessions]…A bigger group would definitely had made it better*.” Participant Oliver |
|  | - Enhance group cohesion across variety of member characteristics | | “*It was difficult to get to know other people…one of them actually said that they couldn’t see that I had problems, [they] struggled with communicating and I don’t in the same way. I mean I do compared to how I was previously. But, [they] found it harder that he couldn’t see anything wrong with me.”* Participant Coleen |
|  | - Dedicated tech support for online sessions | | “*There are clearly some people who are going to find [sessions] much more, much less accessible. Some people are really going to struggle with the technology front.”* Participant Cory |
|  | - Pre-select participants with similar characteristics/ issues | | “*I had nothing in common with my group, which made it difficult to strike up a conversation. I think if the people that was in the group were more my own age, we may have more in common, which would make it easier.”* Participant Arthur |
| **Facilitator Factors** | **Perceived Benefits and Prerequisites for Success** | | |
|  | - Acknowledging individuality of ABI effects | | *“I think the understanding, the understanding of everybody involved is one of the biggest helpful things that actually, you could have a brain injury yourself because of your level of understanding is so good.”* Participant Alison |
|  | - Engaging but not forceful facilitation | | *“I think a good thing was also that [the facilitator] would direct this, although [the facilitator] wouldn’t say ‘this is what you have to be talking about.’ She would start it up and let us go on our own tangents sometimes, which was quite helpful.”* Participant Gillian |
|  | - Being available if needed between sessions | | *“It was always made clear as well, you know, if anything, it’s difficult or upsetting, [the facilitator] said ‘contact me any time, here’s my mobile, email me, call me whenever.’ So [the facilitator] just made it clear that we could contact them with any issue.”* Participant Jane |
|  | **Suggestions for Improvement** | | |
|  | - Ensure equal participation from group members | | *“Instead of there being [all of our] voices, one of them was very loud and dominating. It made me feel that my opinion didn’t count.”* Participant Arthur |

**Table S4.** Example quotes for acceptability themes from qualitative interviews. Pseudonyms are used to present the data.

*Document S4: Supporting Participants with Aphasia*

Here, we summarise qualitative data from participants with aphasia to determine the potential suitability of group BA in these individuals. Three of six participants with aphasia withdrew. One of these three completed a bespoke interview, recorded using contemporaneous notes, whilst the 3 participants that continued completed all assessments. This resulted in data from two participants with fluent aphasia (who provided feedback independently), and two with non-fluent aphasia (who were supported by carers in providing feedback). A summary of recommendations based on this data is in Table S1.

Overall, participants with mild fluent aphasia found their group to be positive and generally did not find their speech to be a major barrier to participation. One participant made no reference to their speech prohibiting enjoyment of the AE group within the PSQ. The second participant noted that the AE Group was preferable given their present difficulties with their speech.

*“For me, because my brain injury is quite recent, I think the activity was better for me... because with my speech, I know it sounds OK at the moment, but I’m really conscientious, conscious of the um, the speaking and the group members. But that was fine, um, so there was not a lot, there was um, doing the activities, was relaxing for me.”* Maya, AE Group

However, if other participants spoke rapidly or were perceived to dominate conversation, this was detrimental.

“*I would, I did find really difficult, because my words and the thinking was difficult...it was difficult when people speak over you when you’re talking, I know it’s difficult online with that anyway, but um, I just feel that little bit, it makes me feel quite small when, when that happens.”* Maya, AE Group

For those with non-fluent aphasia, however, taking part in either group was effortful and individual therapy was preferred. This was influenced by feeling “like a charity case” for requiring communication support relative to other group members, smaller group sizes increasing pressure to contribute, and difficulties engaging in activities with verbal components. One carer noted that the main reason to participate was to contribute to research, rather than any perceived direct benefits.

“*[The participant] joined in, it’s because it can be of some use, their difficulties could be of some use to somebody, you know...I thought, well, it’s all extra practice for him, but he finds it upsetting, but he will do it because it’s useful to somebody”* Carer of Mason, AE Group

Positive comments from those with non-fluent aphasia and carers related mainly to a welcoming atmosphere and friendly group members, with structured turn-taking perceived as useful. Learning how people struggle internally after ABI in comparison to external difficulties like aphasia was viewed as beneficial. Online sessions were preferable due to fatigue associated with travel in person. However, this was not sufficient to improve mood.

“*We have a communication group that [he] has been to a few times before the lockdown and everything happened, which I used to drop him off and leave him there. But no matter how caring they are, he felt left out and couldn’t contribute and because they were a group of people – this will come up [for MAPLES] as well - but he doesn’t go anymore because of that, and also they do it online now. But he can’t join in with that either. That’s a good point [for MAPLES], so it was much more ordered. It wasn’t just a general everybody chat at any random time. It was much more go round the group, which is much easier for him, but still frustrating. So he doesn’t join in with the communication group we have anymore at all because it is more harmful than good. He’s come home upset, you know, and struggling and like, ‘what’s the point, I can’t get anything out anyway.’”* Carer of Mason, AE Group

The two carers of those with non-fluent aphasia felt it beneficial to be present to support communication and one felt it should be mandatory for carers to attend. For the AP group specifically, the carer noted that mainly visual and point-form materials would be best for those with non-fluent aphasia. Further, planning only small and non-verbal activities was important. For the AE Group specifically, the carer felt that interest-based groups (e.g., playing online chess only) for people with communication difficulties only would help reduce stigma. Carers noted benefit in facilitators having knowledge of the ways aphasia affects a person, and explicitly confirm they have understood what participants are trying to say.

|  | **Activity Planning Group** | **Activity Engagement Group** | **Both Groups** |
| --- | --- | --- | --- |
| Both Fluent and Non-Fluent Aphasia | - Group slides short with small amount of information - Avoid academic language - Beginning with small non-communication-based activities | - Selection of visual activities (e.g., colouring, painting) or activities adapted for single-word responses - Less time spent solely on conversation | - Facilitator proficient in understanding variety of effects due to aphasia - Facilitator checks to ensure they have understood what participant has said - Structured turn-taking - Bigger group size to reduce pressure to speak - Group rules for other group members to speak slowly - Online delivery preferable |
| Non-Fluent Aphasia | - All study materials in point-form or picture-based - All participants with similar level of communication difficulties - Audio dictated homework options available | - Interest-based groups for those with communication difficulties only | - Family member or carer involved in at least two group sessions - Additional one-to-one support if other group members do not have communication difficulties |

**Table S5**. Summary of recommendations for adapting the MAPLES intervention groups to support those with aphasia based on *n = 4* participants and two carers.

*Mixed- Effects Models Results*

| **BADS** | $b$ | *SE* | 95% CI | df | *t-*value | Pr(>\|t\|) |
| --- | --- | --- | --- | --- | --- | --- |
| (Intercept) | 16.10 | 4.68 | [7.13, 25.16] | 117.82 | 3.44 | **<0.001***** |
| Time 2 | 10.10 | 3.72 | [3.12, 17.47] | 167.41 | 2.75 | **<0.01***** |
| Time 3 | 9.88 | 3.68 | [2.79, 16.99] | 167.42 | 2.68 | **<0.01***** |
| Group 2 | -2.39 | 3.63 | [-9.43, 4.57] | 168.38 | -0.66 | 0.51 |
| Group 3 | -0.26 | 4.23 | [-8.44, 7.87] | 169.73 | -0.06 | 0.95 |
| Baseline BADS | 0.83 | 0.03 | [0.75, 0.90] | 172.47 | 21.89 | **<0.001***** |
| Time 2 * Group 2 | -3.57 | 5.32 | [-13.83, 6.67] | 167.46 | -0.67 | 0.50 |
| Time 3 * Group 2 | -5.68 | 5.26 | [-15.82, 4.44] | 167.72 | -1.07 | 0.28 |
| Time 2 * Group 3 | -9.33 | 6.27 | [-21.43, 2.74] | 167.65 | -1.48 | 0.13 |
| **HADS-Depression** | $b$ | *SE* | 95% CI | df | *t-*value | Pr(>\|t\|) |
| (Intercept) | 1.54 | 0.57 | [0.44, 2.63] | 86.61 | 2.69 | **<0.001**** |
| Time 2 | -1.45 | 0.61 | [-2.65, -0.26] | 167.54 | -2.35 | **<0.05*** |
| Time 3 | -1.46 | 0.61 | [-2.64, -0.28] | 167.54 | -2.38 | **<0.05*** |
| Group 2 | 0.48 | 0.60 | [-0.68, 1.65] | 168.57 | 0.79 | 0.42 |
| Group 3 | -0.14 | 0.70 | [-1.49, 1.21] | 169.99 | -0.20 | 0.83 |
| Baseline HADS-D | 0.83 | 0.03 | [0.75, 0.90] | 169.81 | 22.40 | **<0.001***** |
| Time 2 * Group 2 | -0.75 | 0.88 | [-2.45, 0.95] | 167.56 | -0.85 | 0.39 |
| Time 3 * Group 2 | 0.07 | 0.87 | [-1.61, 1.75] | 167.54 | 0.08 | 0.93 |
| Time 2 * Group 3 | 3.52 | 1.04 | [1.51, 5.53] | 167.81 | 3.37 | **<0.001***** |
| **HADS-Anxiety** | $b$ | *SE* | 95% CI | df | *t-*value | Pr(>\|t\|) |
| (Intercept) | 0.85 | 0.51 | [-0.13, 1.84] | 94.11 | 1.64 | 0.10 |
| Time 2 | -2.20 | 0.64 | [-3.44, -0.87] | 167.32 | -3.44 | **<0.001***** |
| Time 3 | -1.65 | 0.63 | [-2.87, -0.43] | 167.59 | -2.61 | **<0.01**** |
| Group 2 | 0.24 | 0.61 | [-0.95, 1.43] | 170.92 | 0.38 | 0.69 |
| Group 3 | 0.02 | 0.72 | [-1.37, 1.41] | 172.94 | 0.03 | 0.97 |
| Baseline HADS-A | 0.88 | 0.03 | [0.82, 0.95] | 167.38 | 27.74 | **<0.001***** |
| Time 2 * Group 2 | 2.46 | 0.91 | [0.71, 4.23] | 167.35 | 2.74 | **<0.01**** |
| Time 3 * Group 2 | 1.46 | 0.90 | [-0.28, 3.20] | 167.35 | 1.61 | 0.10 |
| Time 2 * Group 3 | 3.32 | 1.07 | [1.25, 5.41] | 167.72 | 3.08 | **<0.01**** |
| **BMQ-S** | $b$ | *SE* | 95% CI | df | *t-*value | Pr(>\|t\|) |
| (Intercept) | 12.77 | 3.31 | [6.43, 19.28] | 142.79 | 3.85 | **<0.001***** |
| Time 2 | -7.37 | 1.90 | [-11.01, -3.68] | 164.21 | -3.86 | **<0.001***** |
| Time 3 | -8.32 | 1.88 | [-11.94, -4.70] | 164.19 | -4.42 | **<0.001***** |
| Group 2 | 0.68 | 1.81 | [-2.80, 4.21] | 165.27 | 0.37 | 0.70 |
| Group 3 | -0.36 | 2.13 | [-4.46, 3.75] | 166.97 | -0.17 | 0.86 |
| Baseline BMQ-S | 0.84 | 0.03 | [0.77, 0.91] | 164.19 | 23.44 | **<0.001***** |
| Time 2 * Group 2 | 5.18 | 2.69 | [0.01, 10.37] | 164.26 | 1.92 | 0.05 |
| Time 3 * Group 2 | 4.42 | 2.65 | [-0.68, 9.55] | 164.21 | 1.66 | 0.09 |
| Time 2 * Group 3 | 9.88 | 3.16 | [3.80, 15.99] | 164.57 | 3.12 | **<0.01**** |

| **SCS-PC** | $b$ | *SE* | 95% CI | df | *t-*value | Pr(>\|t\|) |
| --- | --- | --- | --- | --- | --- | --- |
| (Intercept) | 5.91 | 1.76 | [2.51, 9.30] | 172 | 3.44 | **<0.01**** |
| Time 2 | -2.36 | 1.63 | [-5.49, 0.89] | 172 | -1.44 | 0.14 |
| Time 3 | -2.20 | 1.61 | [-5.30, 0.89] | 172 | -1.36 | 0.17 |
| Group 2 | 1.01 | 1.58 | [-2.02, 4.04] | 172 | 0.63 | 0.52 |
| Group 3 | 0.38 | 1.84 | [-3.15, 3.91] | 172 | 0.20 | 0.83 |
| Baseline SCS-PC | 0.81 | 0.04 | [0.73, 0.89] | 172 | 18.75 | **<0.001***** |
| Time 2 * Group 2 | 2.09 | 2.32 | [-2.37, 6.56] | 172 | 0.90 | 0.36 |
| Time 3 * Group 2 | 2.02 | 2.31 | [-2.41, 6.47] | 172 | 0.87 | 0.38 |
| Time 2 * Group 3 | 2.18 | 2.74 | [-3.08, 7.44] | 172 | 0.79 | 0.42 |
| **SCS-PM** | $b$ | *SE* | 95% CI | df | *t-*value | Pr(>\|t\|) |
| (Intercept) | 5.00 | 1.30 | [2.48, 7.49] | 135.73 | 3.83 | **<0.001**** |
| Time 2 | 0.87 | 0.98 | [-1.01, 2.76] | 166.59 | 0.88 | 0.37 |
| Time 3 | 0.32 | 0.97 | [-1.54, 2.20] | 166.52 | 0.33 | 0.73 |
| Group 2 | -0.75 | 0.95 | [-2.59, 1.09] | 169.77 | -0.78 | 0.43 |
| Group 3 | -0.05 | 1.11 | [-2.18, 2.07] | 171.19 | -0.05 | 0.96 |
| Baseline SCS-PM | 0.75 | 0.05 | [0.64, 0.86] | 159.72 | 13.62 | **<0.001***** |
| Time 2 * Group 2 | 0.39 | 1.40 | [-2.30, 3.09] | 166.51 | 0.28 | 0.77 |
| Time 3 * Group 2 | 0.39 | 1.39 | [-2.29, 3.07] | 166.55 | 0.28 | 0.77 |
| Time 2 * Group 3 | -3.22 | 1.65 | [-6.40, -0.03] | 167.45 | -1.94 | 0.05 |
| **IU-PA** | $b$ | *SE* | 95% CI | df | *t-*value | Pr(>\|t\|) |
| (Intercept) | 5.17 | 1.19 | [2.88, 7.46] | 116.37 | 4.32 | **<0.001***** |
| Time 2 | 0.07 | 1.02 | [-1.90, 2.05] | 167.25 | 0.07 | 0.94 |
| Time 3 | -1.06 | 1.01 | [-3.02, 0.89] | 167.26 | -1.04 | 0.29 |
| Group 2 | 0.13 | 0.99 | [-1.76, 2.05] | 167.91 | 0.13 | 0.89 |
| Group 3 | -0.12 | 1.16 | [-2.36, 2.13] | 169.77 | -0.11 | 0.91 |
| Baseline IU-PA | 0.75 | 0.04 | [0.66, 0.83] | 172.53 | 17.21 | **<0.001***** |
| Time 2 * Group 2 | 2.34 | 1.46 | [-0.47, 5.18] | 167.29 | 1.59 | 0.11 |
| Time 3 * Group 2 | 2.16 | 1.45 | [-0.63, 4.96] | 167.28 | 1.48 | 0.13 |
| Time 2 * Group 3 | -1.50 | 1.73 | [-4.83, 1.83] | 167.49 | -0.86 | 0.38 |
| **IU-IA** | $b$ | *SE* | 95% CI | df | *t-*value | Pr(>\|t\|) |
| (Intercept) | 3.13 | 1.03 | [1.16, 5.13] | 144.20 | 3.03 | **<0.01***** |
| Time 2 | -2.91 | 0.91 | [-4.67, -1.16] | 167.06 | -3.20 | **<0.01***** |
| Time 3 | -2.63 | 0.90 | [-4.36, -0.89] | 167.09 | -2.92 | **<0.01***** |
| Group 2 | 0.36 | 0.87 | [-1.32, 2.06] | 167.81 | 0.41 | 0.67 |
| Group 3 | -0.26 | 1.03 | [-2.28, 1.71] | 171.32 | -0.25 | 0.79 |
| Baseline IU-IA | 0.78 | 0.05 | [0.67, 0.88] | 171.30 | 14.24 | **<0.001***** |
| Time 2 * Group 2 | 2.19 | 1.30 | [-0.30, 4.70] | 167.13 | 1.68 | 0.09 |
| Time 3 * Group 2 | 1.34 | 1.28 | [-1.13, 3.81] | 167.12 | 1.04 | 0.29 |
| Time 2 * Group 3 | 4.72 | 1.53 | [1.77, 7.67] | 167.47 | 3.08 | **<0.01**** |

**Table S6.** Full results of mixed effects models.

BADS= Behavioural Activation for Depression Scale, HADS = Hospital Anxiety and Depression Scale, BMQ-S = Brain Injury Rehabilitation Trust Motivation Questionnaire-Self; SCS-PC = Sense of Control Scale-Perceived Constraints; SCS-PM = SCS-Perceived Mastery; IU-PA = Intolerance of Uncertainty Scale-12-Prospective Anxiety; IU-IA = IUS-12 – Inhibitory Anxiety

****p* <0.001, ***p* < 0.01, **p* <0.05

*Visualisation of Mean Differences for Study Outcomes
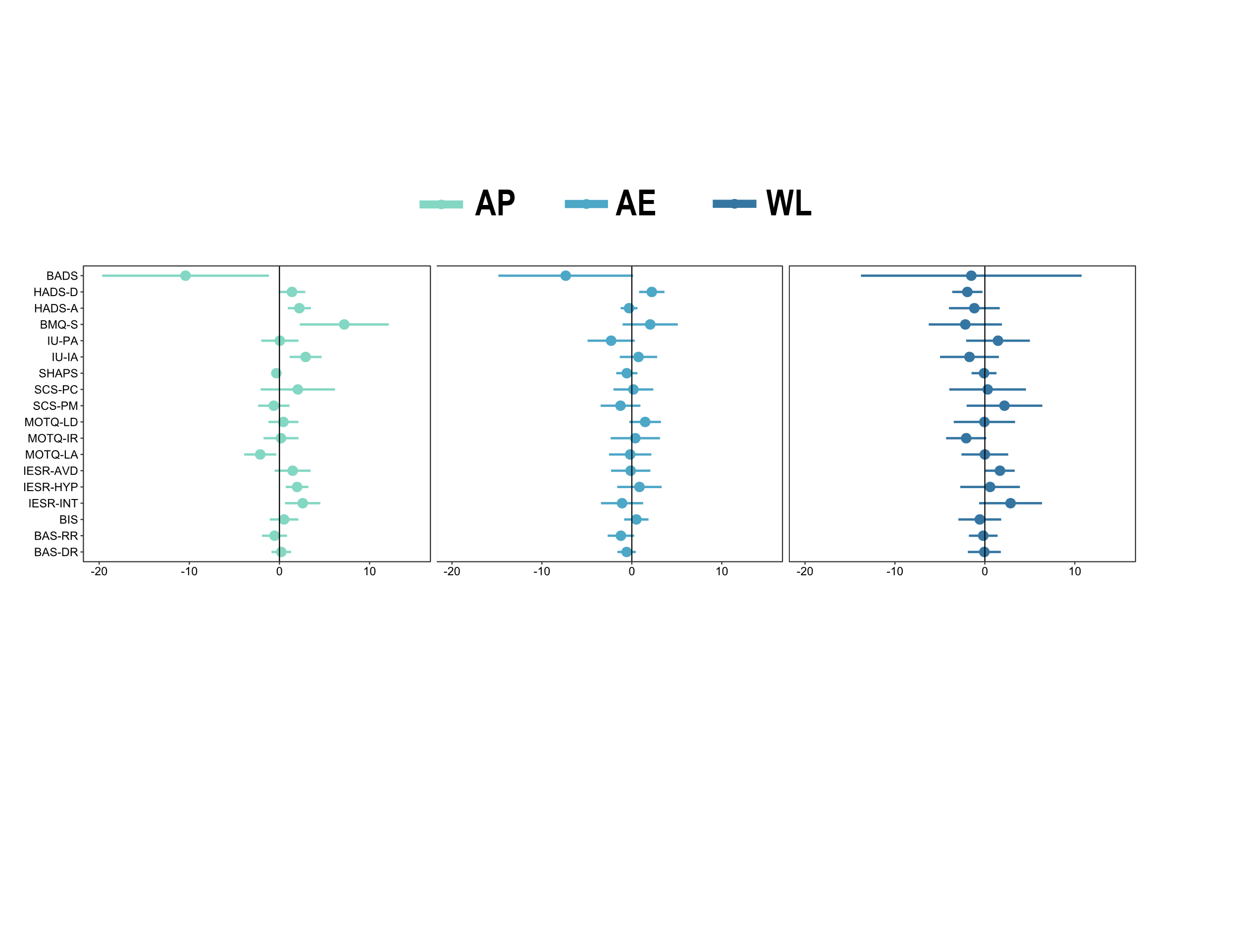
*

**Figure S3.** Visualisation of raw unadjusted mean differences on study outcome measures from baseline to post-intervention. Of note, negative scores on the BADS, all MOT-Q subscales, BAS-Reward Responsiveness, BAS-Drive, SCS-Perceived Mastery, and SHAPS indicate improvement. Positive scores on the HADS subscales, BMQ-S, IES-R subscales, BIS Inhibition, SCS-Perceived Constraints, and IU-SF subscales indicate improvement. Mean differences are plotted without scaling for differences in scale range.

*Note.* AP = Activity Planning Group; AE = Activity Engagement Group; WL = Waitlist; BADS=Behavioural Activation for Depression Scale, HADS=Hospital Anxiety and Depression Scale, HADS-D = HADS-Depression, HADS-A = HADS-Anxiety, BMQ-S = BIRT Motivation Questionnaire-Self, SCS = Sense of Control Scale, SCS-PC = SCS-Perceived Constraints, SCS-PM = SCS-Perceived Mastery, SHAPS=Snaith-Hamilton Pleasure Scale, IU-SF =Intolerance of Uncertainty Scale-Short Form, IU-PA = IU-Prospective Anxiety; IU-IA = IU-Inhibitory Anxiety, MOT-Q = Motivation for Traumatic Brain Injury Rehabilitation Questionnaire, MOTQ-LD = MOT-Q Lack of Denial, IESR-AVD = IES-R Avoidance, IESR-HYP = IESR-Hyperarousal, IESR-INT = IES-R-Intrusions MOTQ-IR = MOT-Q Interest in Rehabilitation, MOTQ-LA = MOT-Q Lack of Anger, IES-R = Impact of Event Scale-Revised, BIS/BAS = Behavioural Inhibition/Behavioural Activation Scales, BAS-RR = BAS-Reward Responsiveness, BAS-DR = BAS Drive

*Clinically Meaningful Improvements on Study Measures*

MCID estimates were not calculated for the MOM-POPS due to its categorical format, nor for the MOT-Q Reliance on Professional Help subscale and the BIS/BAS Fun Seeking subscale due to poor internal consistency (α < 0.60).

| MCID Change – *n* (%) | Activity Planning Group  (*n* = 24) | Activity Engagement Group  (*n* = 23) | Waitlist Controls  (*n* = 13) |
| --- | --- | --- | --- |
| HADS  Depression  Anxiety | 13 (54.17%)  13 (54.17%) | 13 (56.52%)  5 (20.83%) | 1 (7.69%)  2 (15.38%) |
| BMQ-S | 13 (56.52%) | 9 (39.10%) | 3 (23.08%) |
| MOT-Q  Lack of Denial  Interest in Rehabilitation  Lack of Anger | 6 (26.09%)  4 (17.39%)  11 (47.83%) | 3 (13.63%)  5 (22.73%)  4 (18.18%) | 4 (36.36%)  5 (45.45%)  3 (27.27%) |
| IES-R  Avoidance  Hyperarousal  Intrusions | 7 (29.17%)  10 (41.67%)  10 (41.67%) | 5 (21.74%)  6 (26.09%)  5 (21.74%) | 5 (38.46%)  3 (23.08%)  5 (38.46%) |
| BIS/BAS  Inhibition  Reward Responsiveness  Drive | 7 (29.17%)  6 (25.00%)  4 (16.67%) | 6 (26.09%)  8 (34.78%)  9 (39.13%) | 4 (30.77%)  3 (23.08%)  4 (30.77%) |
| SCS  Perceived Mastery  Perceived Constraints | 11 (45.83%)  9 (37.50%) | 10 (43.48%)  4 (17.39%) | 1 (7.69%)  4 (30.77%) |
| SHAPS | 5 (20.83%) | 8 (34.78%) | 4 (30.77%) |
| IU-SF  Prospective Anxiety  Inhibitory Anxiety | 4 (16.67%)  14 (58.33%) | 5 (21.74%)  10 (43.48%) | 4 (30.77%)  5 (38.46%) |

**Table S7.** Summary of the number of participants in each arm of the MAPLES trial that showed improvements by at least the scale-specific minimal clinically important difference (MCID). Percentages were calculated based on those who had provided data at Time 2 (i.e., did not withdraw prior to this point).

BADS=Behavioural Activation for Depression Scale, HADS=Hospital Anxiety and Depression Scale, BMQ-S = BIRT Motivation Questionnaire-Self, MOT-Q = Motivation for Traumatic Brain Injury Rehabilitation Questionnaire, IES-R = Impact of Event Scale-Revised, BIS/BAS = Behavioural Inhibition/Behavioural Activation Scales, SCS = Sense of Control Scale, SHAPS=Snaith-Hamilton Pleasure Scale, IU-SF =Intolerance of Uncertainty Scale-Short Form

***Exploration of Potential Harms***

Harms were operationalised as worsening HADS scores and changes in reporting of suicidality. Of note, participants were not insulated from other events (e.g., COVID-19) and mood fluctuations in both directions were expected.

*Harms – Worsening on Mood Measures*

In the AP and AE Groups, the proportion of those improved was higher than WL using complete cases, suggesting there is likely no disproportionate harm (Supplementary Table 8).

|  | MCID Change –  *n* (%) | Activity Planning Group (*n* = 24) | Activity Engagement Group (*n* = 23) | Waitlist Controls  (*n* = 13) |
| --- | --- | --- | --- | --- |
| HADS-Depression | Improved | 13 (54.2%) | 13 (56.5%) | 1 (7.69%) |
|  | No Change | 7 (29.2%) | 8 (34.8%) | 4 (30.8%) |
|  | Worsened | 4 (16.7%) | 2 (8.7%) | 8 (61.5%) |
| HADS-Anxiety | Improved | 13 (54.2%) | 5 (21.7%) | 2 (15.4%) |
|  | No Change | 9 (37.5%) | 12 (52.2%) | 7 (53.8%) |
|  | Worsened | 2 (8.33%) | 6 (26.1%) | 4 (30.8%) |

**Table S8.** Summary of change as measured by the minimal clinically important difference (MCID) on the HADS-Anxiety and HADS-Depression subscales using complete-case data from Time 1 to Time 2 only.

If a “worst case scenario” sensitivity analysis approach is taken^58^ and assume that AP and AE participants with missing data worsened, and WL participants with missing data improved, AP and AE improvements were still proportionally higher, particularly on HADS-Depression (Supplementary Table 9). However, using this approach, the proportion of AE participants with worsening HADS-Anxiety is potentially concerning.

|  | MCID Change –  *n* (%) | Activity Planning Group (*n* = 29) | Activity Engagement Group (*n* = 28) | Waitlist Controls  (*n* = 16) |
| --- | --- | --- | --- | --- |
| HADS-Depression | Improved | 13 (44.8%) | 13 (46.4%) | 4 (25.0%) |
|  | No Change | 7 (24.1%) | 8 (28.6%) | 4 (25.0%) |
|  | Worsened | 9 (31.0%) | 7 (25.0%) | 8 (50.0%) |
| HADS-Anxiety | Improved | 13 (44.8%) | 5 (17.8%) | 5 (31.3%) |
|  | No Change | 9 (31.0%) | 12 (42.9%) | 7 (43.7%) |
|  | Worsened | 7 (24.1%) | 11 (39.3%) | 4 (25.0%) |

**Table S9.** Summary of change as measured by the minimal clinically important difference (MCID) on the HADS-Anxiety and HADS-Depression subscales, assuming that participants in the AP and AE Group who withdrew from study worsened as a result of the intervention, and Waitlist participants who withdrew improved.

Given mood benefits in either group, using a WL condition, where participants are ensured access to an intervention, is potentially more ethical than a treatment-as-usual control without subsequent treatment allocation.

*Harms – Reporting of Suicidality*

Though participants at immediate risk of self-harm/suicidality were excluded, participants reporting thoughts such as “*I would be better off dead*” but no stated intent to self-harm were not. Including those re-randomised, such thoughts were reported by 13 participants at Time 1 (AP = 3, AE = 6, WL = 4), 9 at Time 2 (AP = 2, AE = 5, WL = 2) and 9 at Time 3 (AP = 4, AE = 5). These were mostly reported as abstract thoughts rather than concrete thoughts of self-harm. In general, there was no evidence that participation had a particular influence on suicidal ideation.

In summary, participation did not appear associated with significant harm, nor systematic worsening in depression or anxiety. However, given the “worst case scenario” sensitivity analysis, exploring whether HADS-Anxiety scores show reliable increases in AE-style groups and whether this is balanced by depression reductions is warranted.

*Document S5: Challenges in Trial Implementation*

*Recruitment*

- Recruiting active NHS participants was challenging, with reasons including concerns about interactions with NHS-based input and finding time to review cases for study referral. Service user groups were good sources of recruitment.
- Recruiting from ABI charities and through social media was efficient, particularly for online intervention delivery.

*Delivering Sessions In-person vs Online*

- Delivery of didactic aspects of AP online was similar to in-person. The AE group was more challenging online than in-person due to having to post materials, and more “stilted” interactions between members using videoconferencing.
- Technical issues interfered with online delivery for both groups. Distractions in the home and, for some, lack of private space to participate, affected conversation. Conversations that may occur before/after in-person groups were absent and including additional unstructured “post-session time” in future online groups may be useful.
- Advantages of online groups included no travel time, participants who may have forgotten sessions joining quickly if prompted, and safety in terms of the pandemic.

*Mixed-Methods Data Collection*

- Collecting both quantitative and qualitative data collection proved challenging when methodologies were at odds with each other (e.g., purposively sampling participants to interview could affect blinded outcomes for quantitative data)
- Collecting qualitative data at both Time 2 and Time 3 may help reduce difficulties with recall in those with ABI.

*Participant Preference in Groups*

- Some participants preferred either the AP or AE Group prior to enrolment – more participants could be recruited if benefits of either group were clearer in study advertising.

*Impact of COVID-19*

- The COVID-19 pandemic and resulting lockdowns, social distancing etc. had a huge impact on the range of activities participants could undertake. The impact on results (e.g., less reinforcement from activities/greater social benefits from the online groups) are hard to gauge.
